# Development of Glycan-masked SARS-CoV-2 RBD vaccines against SARS-related coronaviruses

**DOI:** 10.1371/journal.ppat.1012599

**Published:** 2024-09-26

**Authors:** Zuxin Liang, Chunhui Li, Xiaohua Gong, Guoguo Ye, Yushan Jiang, Huiping Shi, Abid Hussain, Mengyuan Zhao, Mengjun Li, Yuxin Tian, Wei Zhao, Yang Yang, Yuanyu Huang, Chenguang Shen, Minghui Yang

**Affiliations:** 1 BSL-3 Laboratory (Guangdong), Guangdong Provincial Key Laboratory of Tropical Disease Research, School of Public Health; Department of Laboratory Medicine, Zhujiang Hospital; Southern Medical University, Guangzhou, People’s Republic of China; 2 School of Life Science; Advanced Research Institute of Multidisciplinary Science; Key Laboratory of Molecular Medicine and Biotherapy, Beijing Institute of Technology, Beijing, People’s Republic of China; 3 National Clinical Research Center for infectious disease, Shenzhen Third People’s Hospital, Second Hospital Affiliated to Southern University of Science and Technology, Shenzhen, People’s Republic of China; 4 Laboratory Animal Center, Anhui Medical University, Hefei, People’s Republic of China; 5 Key Laboratory of Infectious Diseases Research in South China, Southern Medical University, Ministry of Education, Guangzhou, People’s Republic of China; The University of Texas Medical Branch at Galveston, UNITED STATES OF AMERICA

## Abstract

Emerging and recurrent infectious diseases caused by coronaviruses remain a significant public health concern. Here, we present a targeted approach to elicit antibodies capable of neutralizing SARS-CoV-2 variants and other SARS-related coronaviruses. By introducing amino acid mutations at mutation-prone sites, we engineered glycosylation modifications to the Receptor Binding Domain (RBD) of SARS-CoV-2, thereby exposing more conserved, yet less accessible epitopes. We developed both messenger RNA (mRNA) and recombination subunit vaccines using these engineered-RBDs (M1, M2) and the wild-type RBD as immunogens. The engineered-RBD vaccines elicited robust neutralizing responses against various SARS-CoV-2 variants as well as SARS-CoV and WIV1-CoV, and conferred protection in mice challenged with the XBB.1.16 strain. Furthermore, We highlighted that glycan masking is a decisive factor in antibody binding changes and RBD-conserved antibody response. Additionally, the glycan-engineered RBD mRNA vaccines stimulated stronger cell-mediated immune responses. Our glycan modification strategy significantly enhances broad-spectrum neutralizing efficacy and cellular immunity, providing valuable insights for the development of vaccines against a wide range of SARS-related coronaviruses.

## Author summary

Since late 2020, numerous SARS-CoV-2 variants have emerged across different regions, with the Omicron variant and its subvariants (e.g., BA.5, XBB, JN.1, etc.) posing significant challenges to ongoing efforts in controlling the pandemic. This highlights the urgent need for broadly protective vaccines that target both SARS-CoV-2 variants and other SARS-related coronaviruses that could cause future outbreaks. In this study, we developed a glycan modification strategy based on N-glycosylation by introducing amino acid mutations in the RBD. Using these engineered RBDs (M1, M2) and wild-type RBD as immunogens, we created thermostable mRNA vaccines and recombinant subunit vaccines. Our results demonstrate that these engineered-RBD vaccines elicit potent neutralizing responses against notonly various SARS-CoV-2 variants, but also other coronaviruses like SARS-CoV and WIV1-CoV, surpassing the immune response triggered by the wild-type RBD. Furthermore, glycan-engineered RBD mRNA vaccines stimulated stronger cell-mediated immune responses and provided effective protection against XBB strain challenge in mice. We identified key glycosylation modifications at residues K417, L452, R457, and Q493 that play a critical role in inducing broad-spectrum neutralizing antibodies. In particular, the glycosylation of residues R457 and Q493 was especially effective in enhancing this immune response. These findings offer valuable insights for the design of broad-spectrum vaccines against SARS-related coronaviruses.

## Introduction

The coronavirus disease 2019 (COVID-19) pandemic, caused by severe acute respiratory syndrome coronavirus 2 (SARS-CoV-2), has had a profound global impact, heightening public health concerns and emphasizing the importance of pandemic preparedness [1]. As the pandemic extends into its fourth year, the emergence of the Omicron variant and its subvariants (e.g., BA.5, XBB, JN.1 etc) continues to present significant challenges [2,3]. However, there is still a lack of effective vaccine products against SARS-CoV-2 and its variants. Additionally, other human coronaviruses and unidentified coronaviruses continue to emerge as significant human pathogens. Hence, the development of vaccines capable of inducing broad neutralizing antibodies against SARS-CoV-2 and other SARS-CoV-related coronaviruses is crucial for effective protection.

Glycan masking is a promising strategy in vaccine design, leveraging glycans to shield less important regions and direct the immune system toward highly therapeutic epitopes. Inspired by influenza, HIV, and MERS-CoV, which employed additional glycosylation to evade the immune system [4], this shield-glycan approach is primarily employed to confer immune protection against a broader range of viral strains [5]. Previous studies have demonstrated that glycan engineering of the Spike protein can elicit cross-neutralizing responses against various coronaviruses including PaGX, SHC014, WIV1, and CoV-1, due to increased generation of anti-core RBD-specific antibodies [6]. Conversely, removing glycan shields has been shown to enhance B-cell and T-cell responses against viral variants [7]. Glycan-masking the vaccine antigen by introducing N-linked glycosylation at specific sites redirects the immune cells’ attention towards more conserved and vulnerable regions within the RBD, thereby eliciting a higher and broader immune response [8,9].

Antibodies targeting the RBD have demonstrated efficacy against infection, positioning RBD-based vaccines as promising candidates for generating potent and specific neutralizing antibodies [10–13]. Anti-RBD neutralizing antibodies are categorized into four main classes (Class 1–4) based on the target epitopes in the RBD protein [14,15]. Most SARS-CoV-2 variants exhibit mutations in the receptor-binding site, primarily in Class 1 and Class 2 regions, including key residues such as K417, L452, R457, Q493, etc. Antibodies targeting Class 1 epitope are mainly encoded by IGHV3-53/3-66 genes and is generally sensitive to mutations K417N/T in Beta, Gamma, and Omicron [16]. Those targeted Class 2 antibodies interacts with L452, and antibodies were significantly evaded by BA.4/5 owing to L452R RBD mutations [17]. R457 and Q493 are located in the Class 2 region and are important binding epitope for antibodies such as LY-COV555 and P2B-2F6 [18]. In contrast, broad binding antibodies, like S309 and ADG-20, targeting Class 3/4-sites exhibite slightly lower neutralization potency but broader binding breadth against SARS-CoV-2 variants [16,19]. The epitopes of these antibodies map to more conserved but less accessible regions of the RBD than others. In addition, we noticed that K417 and L452 are relatively close to the epitope of the ADG-20 antibody. The attachment of glycans could potentially block ADG-20 epitope, thereby affecting immunogenicity. Therefore, we designed two proteins: Mutant 1 (M1) primarily masking of four sites (K417, L452, R457, Q493), and Mutant 2 (M2) primarily masking two sites (R457, Q493). Vaccine designs that block easier mutant sites to expose more conserved but less accessible regions through glycosylation of the RBD protein (M1, M2) may induce broadly neutralizing antibodies against variants and potentially emerging sarbecoviruses.

The mRNA vaccines utilizing lipid nanoparticle (LNP) delivery system developed by Moderna and BioNTech/Pfizer have demonstrated high effectiveness and widespread use compared to the traditional vaccines [20–22]. Despite their success, the repertoire of clinically applicable LNP is not yet well established, especially lacking in thermostable lipids. To address this, we designed a series of novel ionizable lipid-like materials, and the optimized LNP formulation exhibited favorable physicochemical properties, thermostability, and efficient RNA transportation efficiency [18,23]. In this study, we evaluated the efficacy of these LNP-mRNA RBD vaccines and recombinant RBD protein subunit vaccines with glycan masking in generating immune responses against SARS-CoV-2 variants and other SARS-related coronaviruses.

## Result

### Rational design of SARS-CoV-2 RBD immunogens

**Fig 1:**
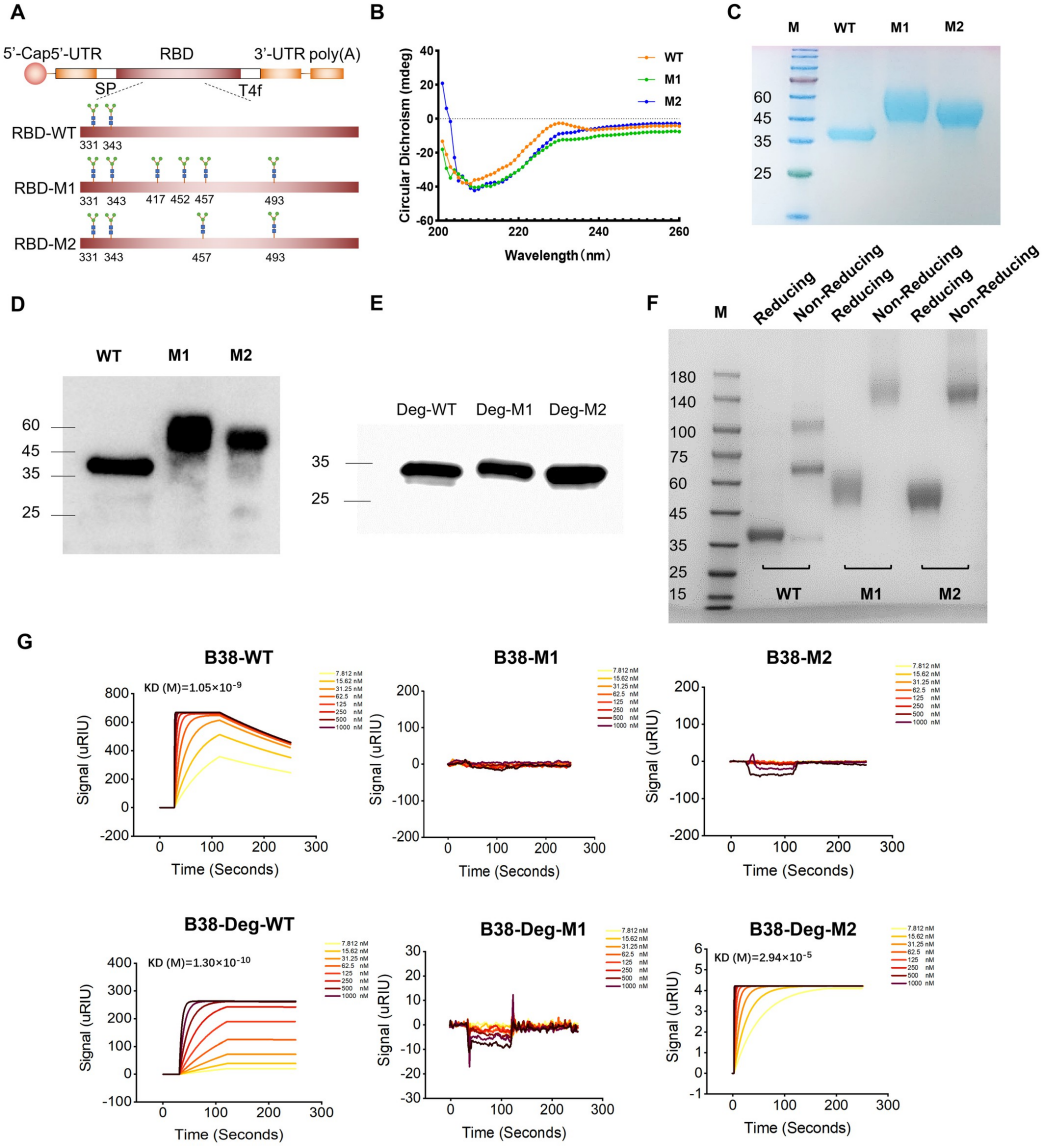
Design and functional analysis of the glycosylated SARS-CoV-2 RBD. (A) Design and construction of three mRNA expressing trimerization RBD with glycosylation. SP: signal peptide; T4f motif: T4 fibritin motif; (B) Circular dichroism of three purified RBD proteins; (C, D) Purified RBD proteins detected by SDS-PAGE and Western blot. (E) Western blot analysis of deglycosylated RBD proteins; (F) Purified RBD proteins detected by reducing and non-reducing page. The left line of each protein was subjected to 4–12% protein gel electrophoresis after boiling in 5×SDS-PAGE loading buffer, which disrupts the trimeric configuration, and the right line of each protein was subjected to 4–12% protein gel electrophoresis after mixed with 5×native sample loading buffer, preserving the protein’s oligomeric structure; (G) The affinity analysis of the monoclonal antibody B38 with different variants of RBD proteins and deglycosylated RBD. WT: wide-type, M1: Mutation 1, M2: Mutation 2.

To develop effective immunogens, we engineered two glycan-modified variants of the SARS-CoV-2 receptor-binding domain (RBD), referred to as Mutation1 (M1) and Mutation2 (M2). To enhance the structural mimicry of the native trimeric RBD, we conjugated the T4f domain (bacteriophage T4 fibritin protein) to the C-terminus of the sequence, promoting the self-assembly of the RBD proteins into trimers. RBD^M1^ incorporated modifications at residues K417N, A419T, L452T, R457N, S459T, T478N, P479A, C480T, E484N, F486T, Q493N and Y495T. RBD^M2^ carried modifications at residues R457N, S459T, T478N, P479A, C480T, E484N, F486T, Q493N and Y495T (Figs 1A and S1). Using endoprotease digestion and liquid chromatography-tandem mass spectrometry (LC-MS/MS) analysis, glycosylation sites and glycan types of engineered RBD protein were identified. The final construct, RBD^M1^, contained six glycan chains at positions N331, N343, K417, L452, R457, and Q493 (S2 Fig and S1 Table), while RBD^M2^ contained four glycan chains at positions N331, N343, R457, and Q493 (S3 Fig and S2 Table). Notably, the mutations at positions T478N and E484N didn not result in glycan modification (S4 Fig).

These glycan-engineered recombinant RBD proteins, along with the wild-type protein, were successfully produced and purified from cell supernatant (Fig 1B and 1C). Western blot analysis confirmed the precise expression of recombinant RBD^M1^ and RBD^M2^ proteins (Fig 1D), with the observed increase in molecular weight attributed to additional glycosylation, validating the successful construction of glycosylated proteins. Subsequent Western blot experiments on deglycosylated proteins confirmed that the increased molecular weight of the two mutant proteins was due to glycan modification (Fig 1E). Non-reducing PAGE analysis further verified the trimeric nature of the proteins, showing that purified RBD^WT^, RBD^M1^, and RBD^M2^ proteins migrated at 110 kDa, 160 kDa, and 150 kDa, respectively. While the RBD^WT^ exhibited a mixture of monomers, dimers, and trimers, M1 and M2 predominantly displayed the trimeric form (Fig 1F). Importantly, surface plasmon resonance (SPR) experiments revealed that glycosylation modifications on the RBD^M1^ and RBD^M2^ proteins hindered their binding with the B38 monoclonal antibody, which primarily recognizes epitopes such as K417, L455, R457, F486, and Q493 (Fig 1G). This suggests that glycosylation effectively masks these epitopes.

### *In vitro* characterization of LNP-mRNA vaccine

**Fig 2:**
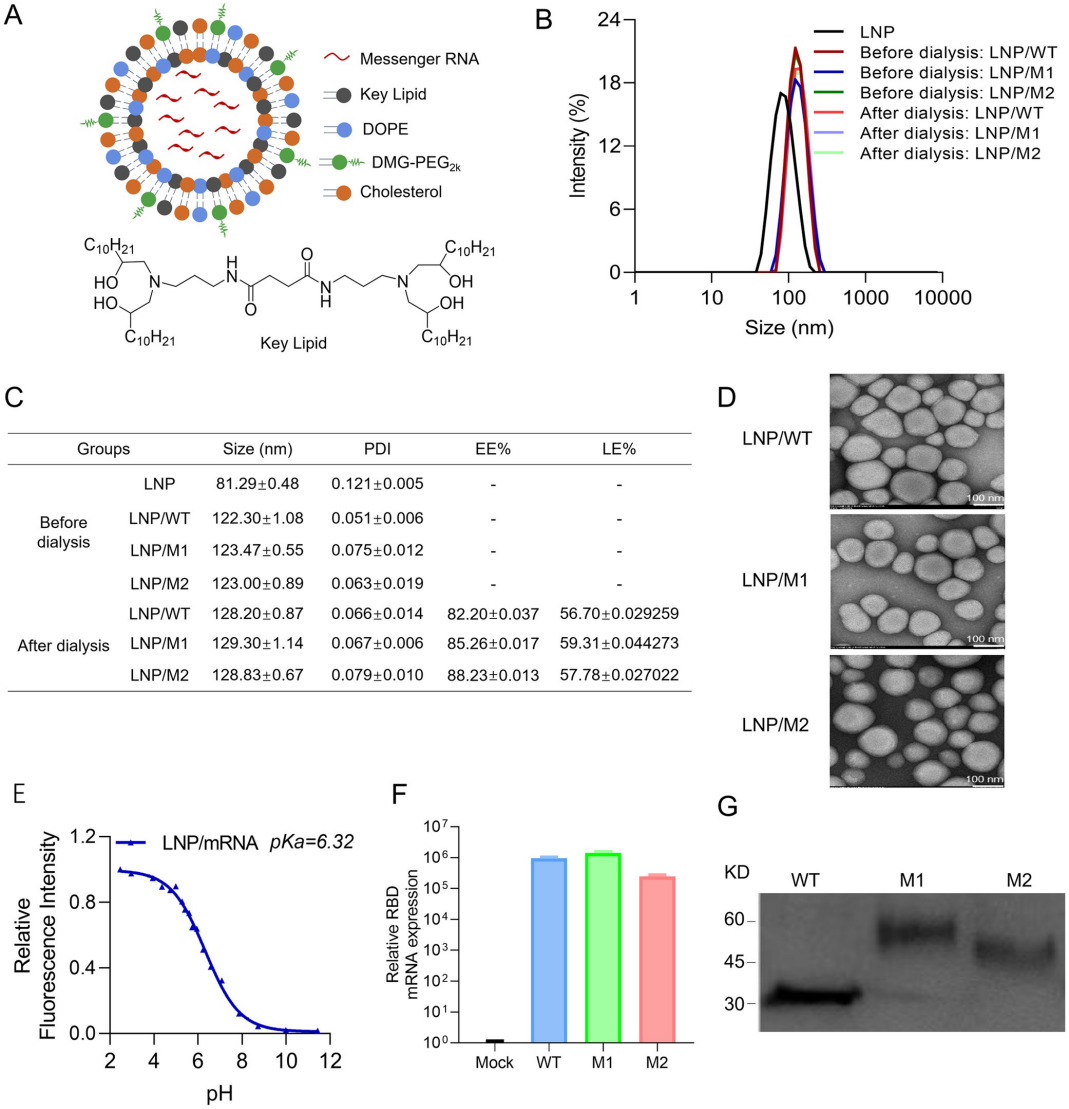
The encapsulation of mRNA vaccine and *in vitro* characterization. (A) Schematic diagram of the LNP-mRNA vaccine; (B) Particle size analysis of LNP-mRNA vaccine before and after dialysis; (C) Particle size, PDI statistics, detection of encapsulation and loading rates of LNP-mRNA complexes; (D) TEM image of LNP-mRNA vaccine. Scale bar, 100 nm; (E) The pKa of LNP-RBD^WT^ mRNA. (F) mRNA expression was detected after plasmid transcription in HEK-293T cells via qRT-PCR. (G) Proteins were expressed in HEK-293T cells followed determined by Western blot.

We developed a vaccine strategy using modified mRNA encapsulated in LNP for efficient antigen delivery. The LNP formulation consists of four lipids: a thermostable key lipid, cholesterol, DOPE, and DMG-PEG_2k_, which self-assemble (Fig 2A). These particles consistently exhibited a uniform sizes, averaging approximately 130 nm, with a polydispersity index (PDI) consistently below 0.15. High mRNA encapsulation efficiency and loading rate were evident from Fig 2B and 2C. Transmission electron microscopy (TEM) further confirmed the spherical shape of LNP-mRNA, corroborating dynamic light scattering (DLS) measurements (Fig 2D).

Previous studies have shown that LNP with an optimal pKa range of 6.0 to 6.5, facilitates rapid ionization within endosomes. This ionization promotes interaction with anionic lipids, such as phosphatidylserine, leading to endosomal membrane destabilization and the subsequent release of nucleic acids into the cytoplasm [22,24]. In our study, the LNP-mRNA exhibited a pKa of 6.32, indicating a positive charge in environments with a pH lower than 6.32, which is conducive to mRNA escape from endosomes (Fig 2E). Three synthetic plasmids were successfully transfected into Human embryonic kidney (HEK)-293T cells using Lipo2000. The presence of mRNA was detected in cells 24 hours post-transfection (Fig 2F), and Western blot analysis confirmed the translation of engineered mRNA vaccines into proteins at the cellular level (Fig 2G). To validate the transcytosis capability of LNP-mRNA, we conducted a coverslip transfer experiment, which demonstrated intercellular transfer of nanoparticles across distinct coverslips. This capability was further validated through flow cytometry (FCM) and confocal microscopy (CLMS) (S5 Fig), highlighting the potential of LNP-mRNA to induce immune responses in both deep and superficial tissues.

### Thermostability assessment of mRNA vaccine under varied temperature conditions

To demonstrate the thermal stability of our LNP system, we used a luciferase reporter mRNA formulation (LNP-Luc-mRNA) to evaluate the storage capability of the mRNA vaccine under different temperature conditions. The LNP-Luc-mRNA formulation was stored at 4°C, 25°C, and 37°C for 1, 3, and 7 days, and then administered intramuscularly (i.m.) to mice. Bioluminescence imaging (BLI) was performed *in vivo* and *in vitro* at 6h, 24h, and 48h post intramuscular administration.

**Fig 3:**
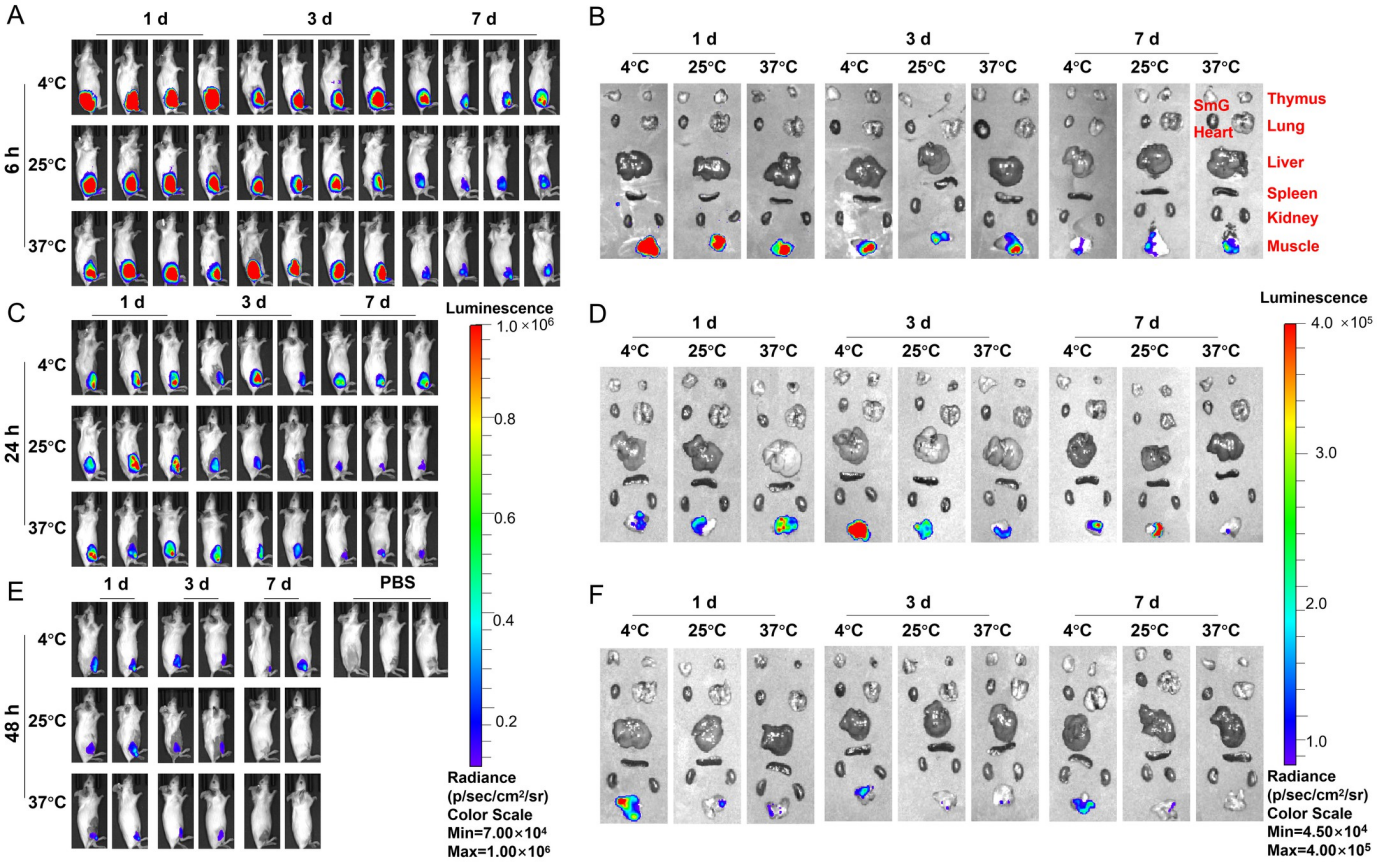
Thermostability of LNP-mRNA formulations under different temperatures. Fluc-encoding reporter LNP-mRNA was stored at 4°C, 25°C and 37°C for 1 d, 3 d, 7 d, and luciferase expression was detected by BLI in vivo and tissues in mice immunized i.m. for (A, B) 6 h (C, D) 24 h and (E, F) 48 h mice. SmG: submandibular gland.

As shown in Fig 3, the expression of Luc-mRNA remained relatively stable even after 3 days of storage at 37°C, with similar results observed at 4°C and 25°C (Fig 3). Additionally, Luc-mRNA was detected exclusively in muscle tissue, with no signals observed in the liver or other organs, indicating that our LNP system effectively minimized liver toxicity while providing a safe and efficient method for antigen delivery.

Serum samples were collected 24 hours after the initial mRNA vaccination with RBD^WT^, RBD^M1^ and RBD^M2^ to measure cytokine levels related to the innate immune response. Levels of IL-4, IL-6, IL-10, IL-12, IL-1β, IFN-γ, and TNF-α in the serum of immunized and naive mice were assessed. The results showed no significant differences in cytokine levels between the immunized and naive mice at 24 hours (S6 Fig), indicating that the administration of LNP-mRNA did not trigger an inflammatory response.

### Evaluation of Glycosylated RBD mRNA vaccines for neutralizing antibodies induction against SARS-CoV-2 variants and protection against the XBB strain

**Fig 4:**
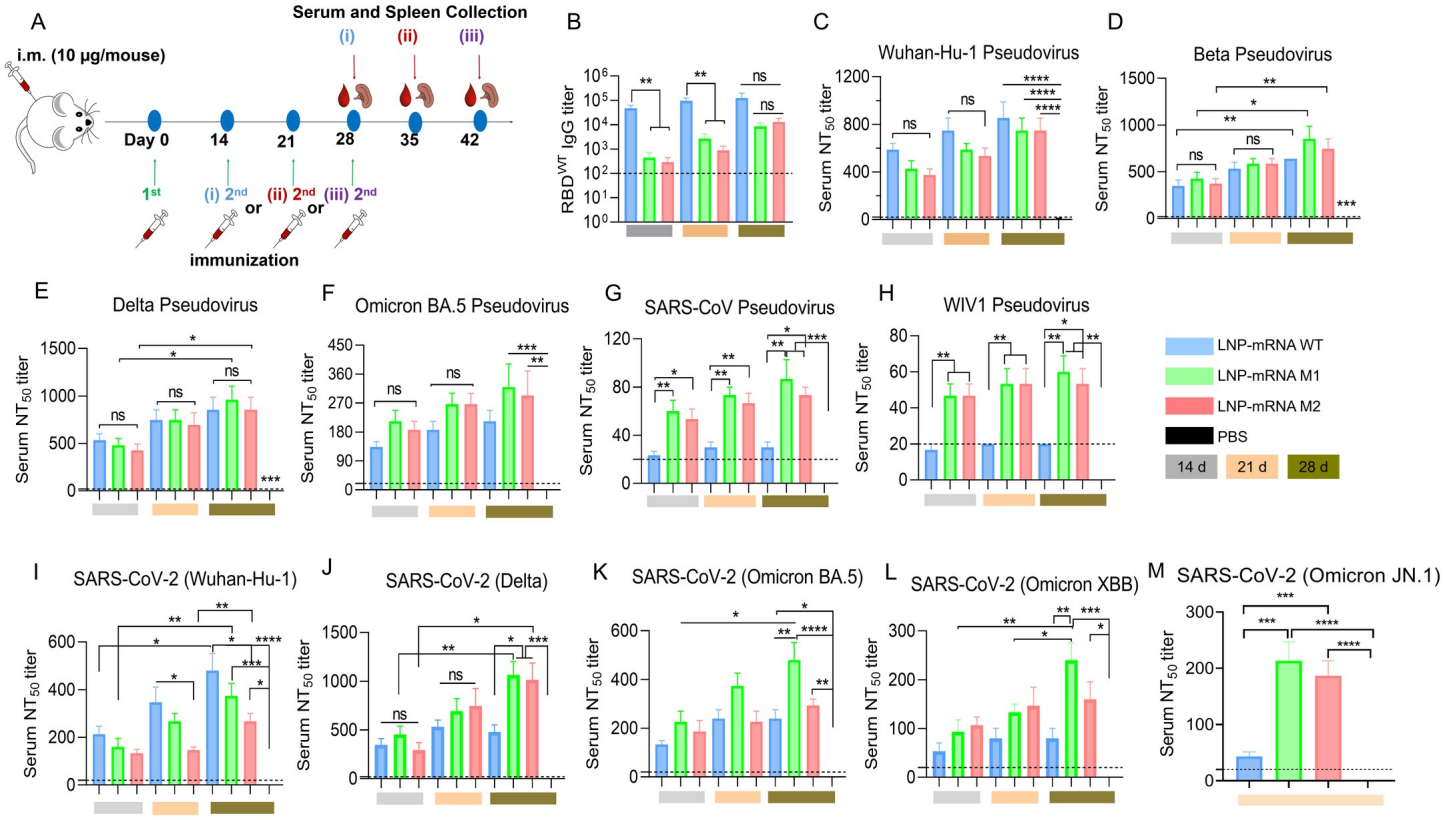
Humoral immune response in vaccinated mice. (A) Schematic diagram of immunization, sample collection and antibody detection. Booster vaccination was administrated on days 14 (i), 21 (ii) and 28 (iii) after the primary immunization and serum was collected two weeks later. (B) Anti-SARS-CoV-2 wide-type RBD IgG titers. (C-H) Neutralization antibody of pseudovirus variants is shown with (C) Wuhan-Hu-1, (D) Beta, (E) Delta, (F) Omicron, BA.5 (G) SARS-CoV, (H) WIV1-CoV. (I-M) Neutralization antibodies against live SARS-CoV-2 Wuhan-Hu-1, Delta, Omicron BA.5, XBB and JN.1 strain. The black dashed line represents the limit of detection (LOD). Statistical significance was determined by one-way ANOVA and two-way ANOVA for comparisons of more than two groups with two or more items. (“*” represents p < 0.05, “**” represents p < 0.01, “***” represents p < 0.001, “****” represents p < 0.0001, “ns” represents not significant).

To evaluate the immune response in mice, BALB/c mice were immunized with 10 μg of mRNA vaccines. Specifically, we investigated the effect of the interval between two immunizations on the immune response with booster shots administered on days 14, 21, and 28 following the initial injection. Serum samples were collected 14 days after the second dose for antibody analysis (Fig 4A).

ELISA results showed that RBD-specific IgG titers were significantly higher in the WT group compared to the M1 and M2 groups (Fig 4B). This difference is likely due to glycosylation masking certain epitopes, leading to reduced antibody recognition of the wild-type RBD protein. However, as the interval between booster doses increased, antibody titers in the M1 and M2 groups also increased, eventually matching those in the WT group. Extending the interval (28 days in this study) between doses mitigated the reduced titers caused by glycosylation.

Pseudovirus neutralization assays confirmed that all mRNA vaccines induced neutralization against SARS-CoV-2 variants, as well as SARS-CoV-1 and WIV1. Although the NT_50_ titers against SARS-CoV-2 Wuhan-Hu-1 were slightly lower for RBD^M1^ and RBD^M2^ compared to WT, they increased with longer dosing intervals, consistent with the ELISA results (Fig 4C). All three vaccines showed similar neutralization effects against Beta and Delta variants, with NT_50_ titers rising as the injection interval was extended (Fig 4D and 4E). Notably, RBD^M1^ and RBD^M2^ induced higher antibody responses against Omicron BA.5 compared to RBD^WT^ (Fig 4F). For SARS-CoV and WIV-1, NT_50_ titers were significantly higher in the RBD^M1^ and RBD^M2^ groups than in the WT group at 21- and 28- days intervals (Fig 4G and 4H). The neutralization assay using live SARS-CoV-2 Wuhan-Hu-1 virus reflected similar trends to the pseudovirus and ELISA results (Fig 4I). Importantly, RBD^M1^ and RBD^M2^ vaccines generated significantly higher antibody responses against Delta, Omicron BA.5, XBB.1.16, and JN.1 strains of SARS-CoV-2 compared to the RBD^WT^ vaccine (Fig 4J–4M). However, it is worth noting that varying the intervals between immunizations has a significant impact on the immune response, suggesting that adjusting dosing intervals could play a crucial role in optimizing overall immune outcomes.

**Fig 5:**
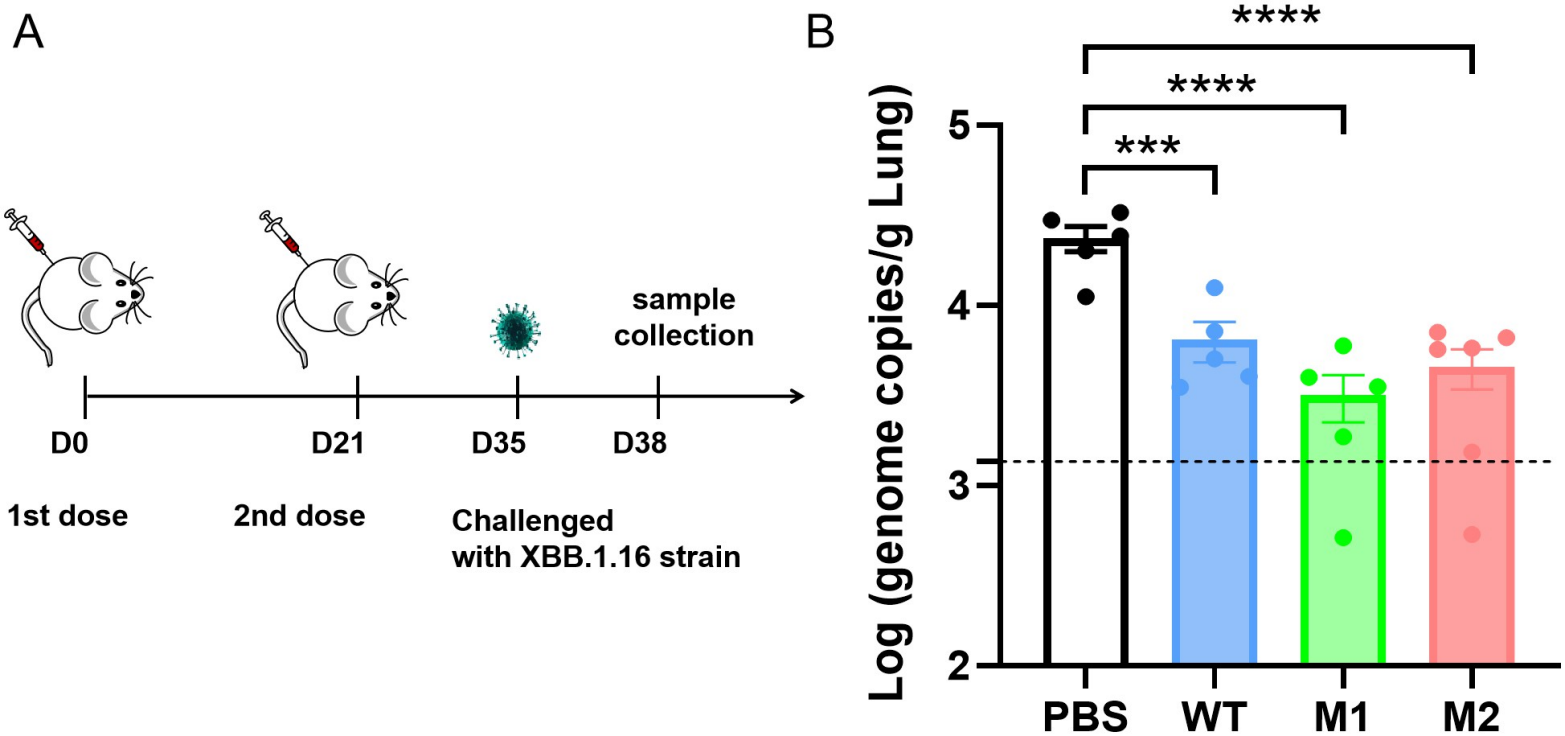
Protection efficiency of the mRNA vaccines in a SARS-CoV-2 XBB challenge mouse model. (A) Schematic of the experimental design, (B) Viral genome copies and SARS-CoV-2 titers in the lung at 3 d.p.i. were measured by qPCR. Statistical significance was determined by one-way ANOVA. (“*” represents p < 0.05, “**” represents p < 0.01, “ns” represents not significant).

To assess the protective capacity of these mRNA vaccines against SARS-CoV-2 *in vivo*, a XBB.1.16 strain challenge mouse model was used. BALB/c mice were immunized with mRNA vaccines at 21-day intervals. 14 days after the second dose, mice were challenged with an intranasal administration of 5×10^5^ TCID50 of the XBB.1.16 strain. 3 days post-challenge, viral loads in the lung tissues were measured via qPCR. The results showed that all three mRNA vaccines significantly reduced viral loads in the lungs of mice (Fig 5). The M1 and M2 vaccine groups echibited a more pronounced reduction in viral loads compared to the PBS group, demonstrating the broad-spectrum immune protection provided by the glycosylated vaccines. Due to the relatively mild virulence of the XBB.1.16 strain in mice, no significant weight loss or lung pathology was observed, only slight pulmonary congestion was noted in the PBS group (S7 Fig).

### Glycan masking as a determinant of antibody binding changes and RBD-conserved immune response

**Fig 6:**
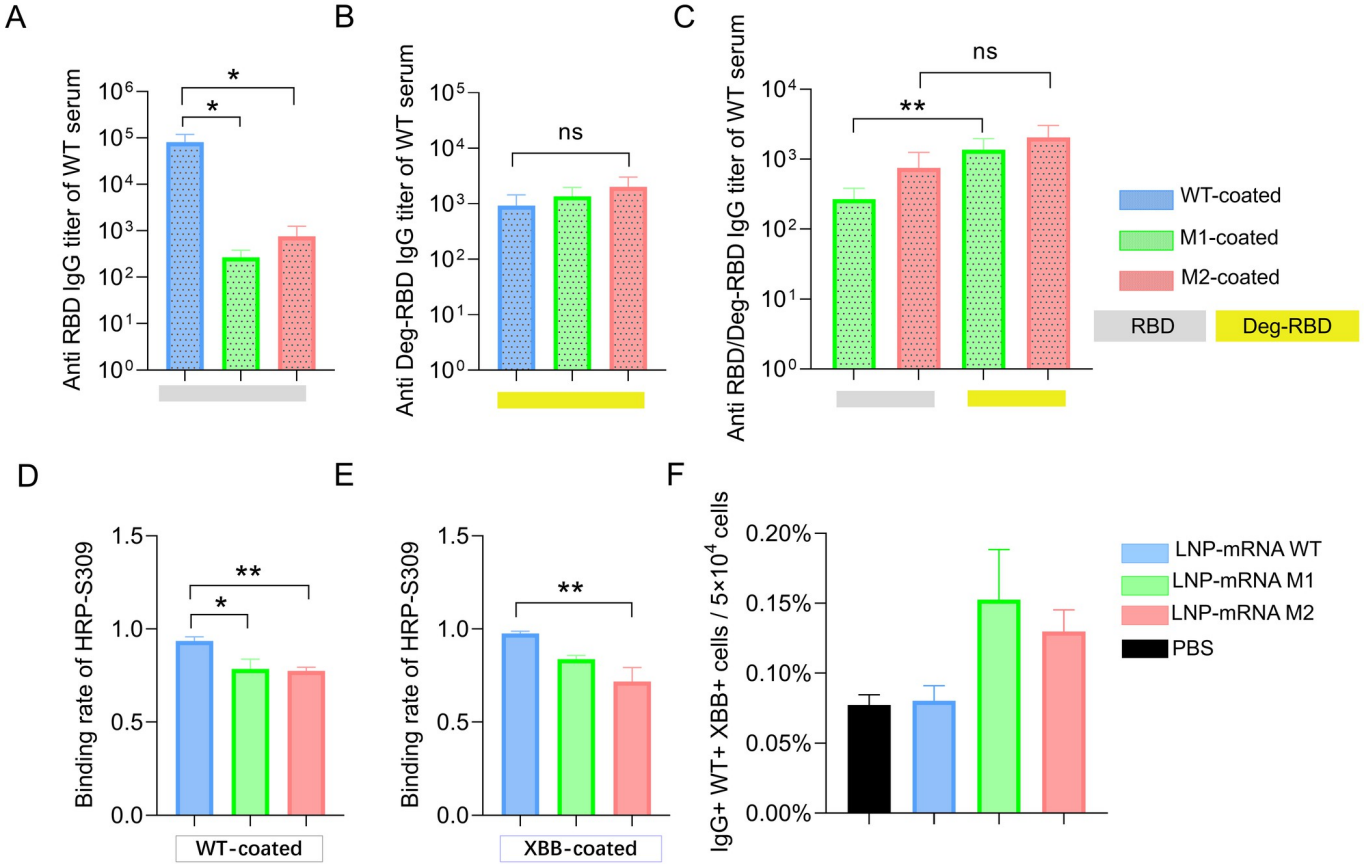
Analysis of RBD-mRNA immunized serum and splenocyte. (A-C) ELISA analysis of RBD^WT^ immunized serum on glycosylated and deglycosylated WT, M1, and M2 coated. (D-E) Antibody competition ELISA of RBD^WT/M1/M2^ immunized serum with WT and XBB as the coating antigen. (F) The representative IgG+ B cells from splenocyte after immunization are shown. Cells were gated for antigen-binding cells (IgG+ WT+ XBB+). The graph shows the percentage of double positive IgG cells for RBD^WT^ and RBD^XBB^ binding. Statistical significance was determined by one-way ANOVA or unpaired, two-sided Student’s t-test for two-group comparisons. (“*” represents p < 0.05, “**” represents p < 0.01, “ns” represents not significant).

To establish glycan masking as the key factor influencing antibody binding variations, we conducted ELISA analysis with plates coated with RBD^WT^, RBD^M1^, and RBD^M2^ proteins. The results showed that serum from WT-immunized mice exhibited significantly higher binding titers to RBD^WT^ compared to RBD^M1^ and RBD^M2^ (Fig 6A). To determine whether the reduced binding of RBD^WT^-immunized serum to RBD^M1^ and RBD^M2^ was due to the presence of surface glycan or the substitution of immunodominant amino acids with asparagine, RBD^M1^ and RBD^M2^ protein were deglycosylated with PNGase F to remove N-linked glycans (Fig 1E). Subsequent ELISA analysis demonstrated similar binding titers of RBD^WT^-immunized serum to RBD^WT/M1/M2^ proteins, suggesting that asparagine substitution did not alter the immune response potency of RBD^WT^-immunized serum (Fig 6B). Additionally, the binding titers of RBD^WT^-immunized serum against deglycosylated RBD^M1^ and RBD^M2^ were higher than those observed before deglycosylation, indicating that epitopes previously obscured by surface glycans became more accessible after glycans removal (Fig 6C).

A competitive ELISA was conducted where S309-HRP protein was mixed with vaccinated serum and added to antigen-coated wells. The binding rate was determined by comparing the OD values of each serum group to the PBS control, thereby eliminating serum background interference. The results revealed decreased binding rates for both M1 and M2 compared to WT (Fig 6D and 6E). This reduction suggests that antibodies in the M1 and M2 immunized sera share overlapping epitopes with S309, a Class 3 antibody. We hypothesize that the enhanced neutralizing ability of M1 and M2 immunized sera against SARS-CoV-2 variants stems from their ability to generate more antibodies targeting conserved epitopes within the Class 3 region.

To explore whether the immune response targeted common epitopes shared by WT and XBB in glycan-modified proteins, we examined IgG^+^ cell responses. FCM analysis showed that approximately 0.153% of IgG^+^ cells derived from the splenocytes of M1-immunized mice bound to both WT and XBB probes, compared to 0.130% in M2 and 0.080% in the WT group (Fig 6F). These findings indicate that glycan modification directs immune responses in mice towards unshielded regions, making conserved and relatively subdominant epitopes more immunogenic, thereby inducing antibodies with cross-reactivity against multiple variants.

### Glycosylated RBD mRNA vaccines elicited SARS-CoV-2-specific T-cell immune responses

**Fig 7:**
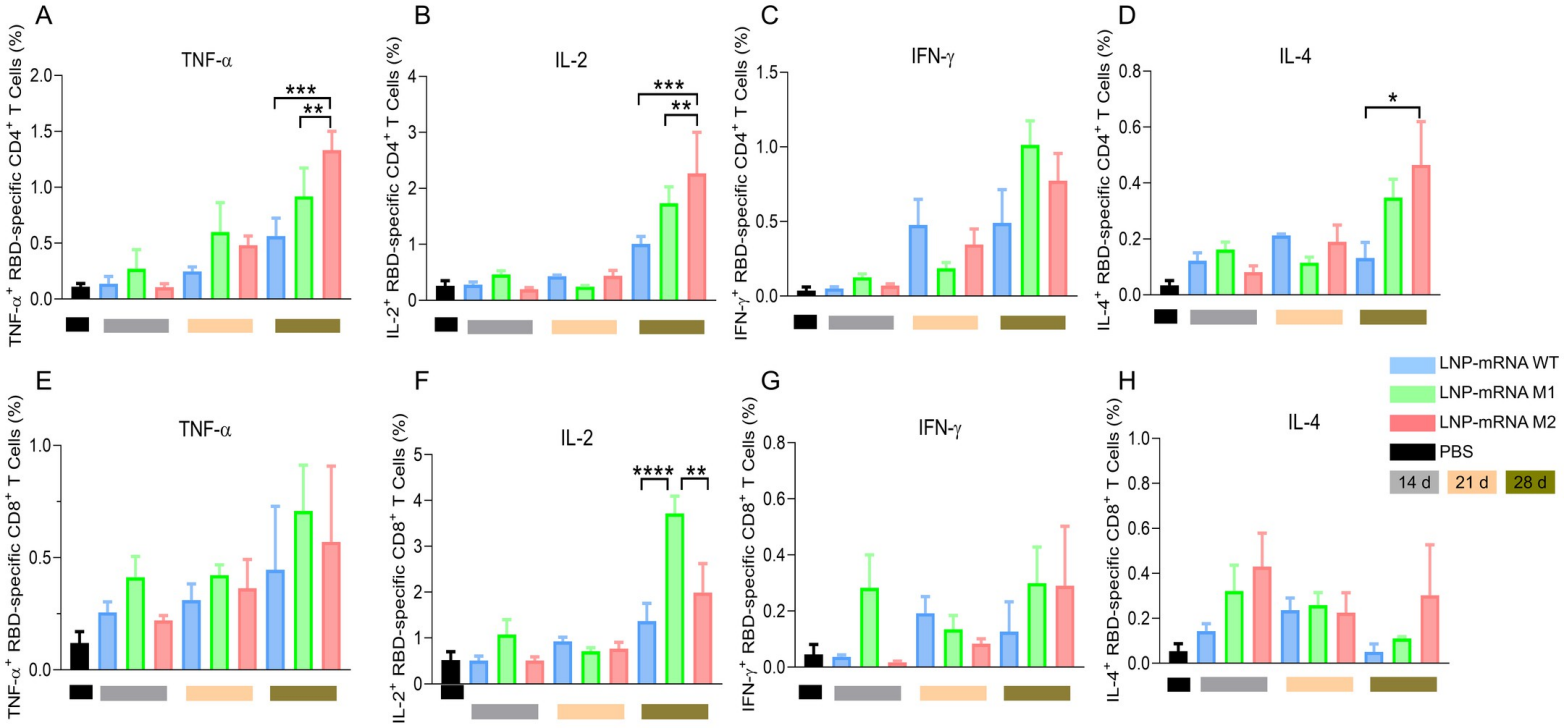
SARS-CoV-2-specific T cell immune response in mRNA vaccinated mice after stimulation of WT RBD-specific peptide pools. (A-D) FCM analysis results show the percentages of CD4^+^ Tem cells secreting (A) TNF-α, (B) IL-2, (C) IFN-γ, and (D) IL-4. (E-H) FCM analysis results show the percentages of CD8^+^ Tem cells secreting (E) TNF-α, (F) IL-2, (G) IFN-γ, and (H) IL-4. Statistical significance was determined by one-way ANOVA. (“*” represents p < 0.05, “**” represents p < 0.01, “***” represents p < 0.001, “****” represents p < 0.0001).

Following booster immunization with the mRNA vaccines, splenocytes were collected from mice and stimulated with SARS-CoV-2 RBD^WT^-specific peptide pools for 8 hours. FCM analysis was conducted to measure the secretion of intracellular cytokines IFN-γ, TNF-α, IL-2, and IL-4. The results revealed a significant enhancement in cell-mediated immune responses, particularly when the interval between the two immunizations was extended (Fig 7). The M1 and M2 groups exhibited stronger responses than the WT group, with notable increase in TNF-α, IL-2, and IL-4 secretion among CD4^+^ T cells. Additionally, the M1 group exhibited significantly higher IL-2 secretion in CD8^+^ T cells compared to the other groups. Overall, the two-dose regimen of the glycosylated mRNA vaccine elicited a robust immune response in SARS-CoV-2-specific helper T cells and cytotoxic T cells, enhancing the immune defense against SARS-CoV-2.

### Glycosylated RBD recombinant subunit vaccines also elicited high levels of broad-spectrum neutralizing antibodies against SARS-CoV-2 variants

**Fig 8:**
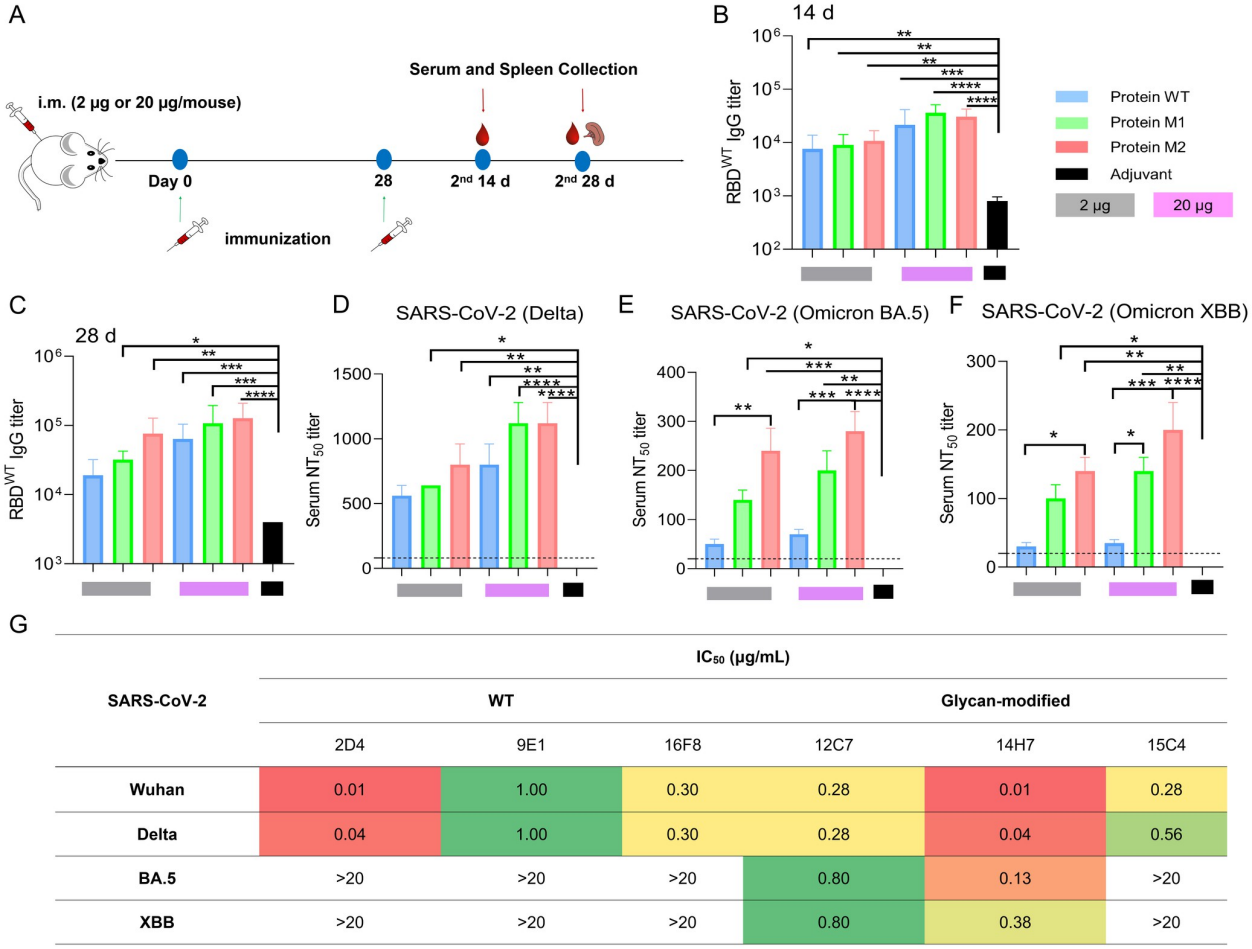
Humoral immune response in recombinant subunit protein-vaccinated mice. (A) The immunization schedule for injection and sample collection. (B, C) Endpoint IgG titers against SARS-CoV-2 RBD were detected in serum collected at day 14 and 28 post-boost immunizations by ELISA. The adjuvant used in this study is the same volume Alum adjuvant, LOD = 100. (D-F) The 50% neutralization titer against live SARS-CoV-2 VOCs (Delta, Omicron BA.5, Omicron XBB strain). Serum was collected in 28 days post-booster immunizations. (G): Authentic neutralizing activity of monoclonal antibodies against SARS-CoV-2 and its variants. Statistical significance was determined by one-way ANOVA and two-way ANOVA for comparisons of more than two groups with two or more items. (^“^*^”^ represents p < 0.05, ^“^**^”^ represents p < 0.01, ^“^***^”^ represents p < 0.001, ^“^****^”^ represents p < 0.0001).

The glycan-marked recombinant proteins were evaluated as antigens in mice. Mice received two doses (2 μg and 20 μg in 50 μL PBS) of the RBD protein vaccines, combined with equal volumes of Alum adjuvants, on days 0 and 28 (Fig 8A). ELISA analysis of serum samples showed a significant rise in RBDWT-specific IgG levels, particularly 14 days after the second dose, with even higher titers by day 28 (Fig 8B and 8C). All three RBD protein vaccines induced neutralizing activity against several live SARS-CoV-2 variants, including Delta, Omicron BA.5 and XBB.1.16 (Fig 8D–8F), consistent with the trends observed for the mRNA vaccines.

To further assess the immune response generated by glycan-modified antigens, six antibodies (9C4, 9B6, 2D4, 3G6, 9E1, 16F8) from the WT group and four antibodies (5C9, 12C7, 14H7, 15C4) from the glycan-modified group was selected for evaluation (S8A Fig). Neutralization assays using authentic virus revealed that antibodies from the WT group were most effective against SARS-CoV-2 Wuhan-Hu-1 and Delta strains, with the antibody 2D4 showing the strongest effect (IC_50_ values of 0.01 and 0.04 μg/mL, respectively). However, these antibodies were less effective against Omicron strains. In contrast, antibodies from glycan-modified group exhibited neutralizing activity against SARS-CoV-2 Wuhan-Hu-1, Delta, Omicron BA.5, and XBB strains. The antibody 14H7 was particularly potent, with IC_50_ values of 0.01, 0.04, 0.13, and 0.38 μg/mL, respectively (Fig 8G). Despite moderate overall binding activity (S8B Fig), these findings suggest that glycan-modified immunization strategies can effectively select broadly neutralizing monoclonal antibodies against SARS-CoV-2 and its variants, providing evidence that glycan design can focus the immune response on more conserved epitope regions.

## Discussion

The emergence of the Omicron variant and its sub-lineages (e.g., BA.5, XBB, JN.1) has highlighted significant challenges in vaccine efficacy due to their varying degrees of antibody evasion [25]. Timely updates to vaccine design are critical for controlling epidemics and reducing mortality rates. The LNP-mRNA platform offers an appealing solution due to its efficacy, rapid preparation, and scalable production, enabling the generation of robust protective responses [26,27]. Our study demonstrates that LNP-mRNA vaccines, administered intramuscularly, maintain effective protein expression and exhibit strong cellular uptake (S5 Fig), ensuring safe and effective antigen delivery. The thermal stability of our vaccine system, which allows for storage at 25°C or 37°C for up to 7 days while retaining protein expression for 6 hours post-administration, is a significant advantage.

Monoclonal antibodies targeting Class1 and Class 2 epitopes (e.g., residues E484, K417, and L452) display a partial or complete loss of efficacy against SARS-CoV-2 variants and show limited cross-reactivity with SARS-related sarbecoviruses [28,29]. In contrast, antibodies targeting conserved Class 3 and Class 4 epitopes, like ADE-20 and S309, exhibit better cross-reactivity against variants [14,30,31]. We specifically targeted glycan modifications at the mutation-prone Class 1 and Class 2 epitopes to focus the immune response on the more conserved Class 3 and Class 4 epitopes, thereby inducing a broader spectrum of immune protection. Notably, glycosylated RBD proteins are more prone to forming stable trimeric structures compared to the wild-type, possibly due to the glycosylation-induced spatial configuration that enhances trimer stability. (Fig 1F). The mRNA-RBD^M1/M2^ vaccines demonstrated superior neutralizing activity against Delta, Omicron BA.5, XBB and JN.1 strains of SARS-CoV-2 compared to the wild-type.

Although the RBD^M2^ subunit vaccine exhibited higher immunogenicity, which differs from the optimal immune response seen with the RBD^M1^ mRNA vaccine, despite there being no significant difference. This suggests that specific modifications, particularly R457N and Q493N, may play a critical role in eliciting a broad-spectrum immune response, while mutations at positions 417 and 452, which are closer to the ADG-20 antibody epitope, might have a lesser impact on immune stimulation. Additionally, the degree of protein glycosylation may vary slightly across different cell lines, potentially leading to minor differences in immunogenicity between mRNA and protein vaccines. It is worth noting that low-dose mRNA vaccines offer superior immune protection compared to protein subunit vaccine. Our results also demonstrate that developing an appropriate immunization strategy, such as adjusting dosing intervals, may have an even more significant impact on the overall immune response.

Glycosylation of target antigens affects antibody response [32], but its effects on T-cell responses remain unclear. In this study, glycosylated RBD mRNA vaccine demonstrated a superior T-cell immune response compared to RBD^WT^, which may be attributed to structural changes in the glycopeptide resulting from glycosylation. Several studies have also shown that glycosylated antigens can significantly enhance T-cell immune responses and accelerate viral clearance [33,34].

There are still several limitations in our study. Firstly, not all amino acid mutations successfully introduced glycan chains. For example, mutations at residues T478N and E484N did not result in glycan modifications, possibly due to steric hindrance caused by the spatial proximity of these two sites and their proximity to the Q493 site. Further research in needed to confirm the effectiveness of glycan modifications through amino acid mutations. Secondly, while engineered antigens demonstrated increased neutralization against some variants compared to wild-type, their neutralization efficacy against the Wuhan-Hu-1 virus decreased. This decrease may be less concerning given the current absence of circulating Wuhan-Hu-1.

## Methods

### Ethics statement

All animal experiments in our study were approved and conducted in accordance with the Institutional Animal Care and Use Committee of Beijing Institute of Technology (BIT-EC-0003-M-125).

### Cell and virus

Vero cells, HEK-293T, and 293T-hACE-2 cells were grown in Dulbecco’s modified Eagle medium (DMEM) supplemented with 10% fetal bovine serum (FBS) and maintained in an incubator at 37°C with 5% CO_2_. The SARS-CoV-2 virus and its variants were isolated from Guangdong Provincial Center for Disease Control and Prevention and Shenzhen Third People’s Hospital.

### Preparation of LNP and LNP-mRNA complex

mRNA plasmids were designed based on an RBD sequence from SARS-CoV-2 isolate Wuhan-Hu-1(MN908947.3). The 5’ untranslated regions (UTR) and IL-2 signal peptide (MYRMQLLSCIALSLALVTNS) were added to the plasmid N-terminus, while the 3’ UTR and T4f trimerization motif sequence (GYIPEAPRDGQAYVRKDGEW VLLSTFL) were added to the C-terminus. The sequences of RBD^WT^, RBD^M1,^ and RBD^M2^ are shown in S1 Fig. The RBD mRNA sequences was prepared using T7 RNA polymerase (E2060s, NEB) with the relevant modifications introduced into the nucleotide sequence as prescribed by the manufacturer, as described previously [35].

The LNP consists of key lipids, DOPE, Cholesterol, and DMG-PEG_2k_. A self-assembly process formed the LNP. Briefly, four components were dissolved with ethanol and mixed in molar ratios of 35.1:11.7:52.6:0.6. Then, three volumes of sodium citrate buffer (pH 4.0) were quickly injected into the lipid mixture. The pre-liposomes were added to mRNA with a mass ratio of 15:1 and incubated at 50°C for 20 min. And liposome nanocomplexes were dialyzed in 1×PBS using a 100 KDa dialysis bag.

### Determination of encapsulation efficiency

Binding of mRNA to fluorescent dye RiboGreen can emit fluorescence and be measured in fluorescence intensity by a microplate reader. Encapsulation efficiency was calculated to (LNP-mRNA/total mRNA in liposomes nanocomplexes) ×100%.

### pKa detection of LNP

The ionization properties of LNP were characterized by pKa values. A series of Nanosolutions with pH ranging from 2.0 to 12.0 were prepared, containing 10 mM HEPES (4-(2-hydroxyethyl)-1-piperazineethanesulfonic acid), 10 mM MES, 10 mM ammonium acetate, 130 mM NaCl, and lipid nanoparticles. TNS (2-(p-toluidino)-6-napthalene sulfonic acid, 100 μM) stock solution was added into the nanosolution at a volume ratio of 1:99. Microplate reader detected the samples at an excitation wavelength of 321 nm and an emission wavelength of 445 nm. Finally, the pKa of lipid nanoparticles was calculated by fitting the Henderson-Hasselbalch equation (GraphPad Prism v.8).

### LNP transcytosis assay

HEK-293T cells were incubated in a 24-well plate at 5×10^4^ cells per well. After 24 hours, the cells were transfected with LNP-coated Cy5-labeled nucleic acid (800 ng per well). Subsequently, the cells were co-incubated with untransfected cells in the 24-well plate at 8 hours after washing three times with PBS. After another 8 hours, the cells were washed three times with PBS and subjected to the repeated co-incubation process described above. The cells were collected at an additional 8 hours and washed three times with PBS and detected Cy5 signals in cells using FCM and CLMS.

### BLI for evaluation of thermostability of the LNP/mRNA (Luciferase mRNA)

LNP/mLuc was prepared at 0, 1, 3, and 7 days before injection. And LNP/mLuc was stored at 4, 25, 37°C for 1, 3, and 7 days. BALB/c mice, with 5 in each group, were intramuscularly (i.m.) injected with 0.5 mg/kg of LNP/mLuc, referring to their body weight. BLI imaging was taken at 6 h, 24 h, and 48 h after injection. Additionally, one mouse from each group was dissected at each time point to measure luciferase expression in muscle and other tissues.

### Construction of RBD trimer protein plasmid and protein expression

The plasmid was designed to encode the native SARS-CoV-2 RBD protein (Wuhan-Hu-1, MN908947.3) and engineered RBD protein fused with a T4f trimerization motif sequence and a 6×His tag at the C-terminus. Both plasmids contain the IL-2 signal peptide sequence at the N-terminus. The optimized RBD gene was cloned into the pCAGGS vector with EcoR I and XhoI restriction sites. HEK293T cells were maintained in DMEM supplemented with 10% FBS. The cells were transfected with recombinant plasmid using PEI MW40000 (CAS 49553-93-7). 48 hours later, the supernatant was collected, and centrifuged at 1000×g for 5 min before filtration using a 0.45 μm Membrane Filter (Millipore). The collected supernatant was purified using NI-NTA Agarose Resin (Yeasen). The purified protein was concentrated using Amicon Ultra Centrifugal Filte (Millipore).

### Detection of protein glycosylation modifications using LC-MS/MS

Protein Digestion: Gel slices were cut into three microcentrifuge tubes, washed with 500 μL of 50 mM ammonium bicarbonate/acetonitrile (1:1, v/v) solution until Coomassie blue disappeared, and then incubated in 1 mL of acetonitrile for 30 minutes. After removal of acetonitrile, gel slices were rehydrated in 10 mM DTT/50 mM ammonium bicarbonate and incubated at 56°C for 1 hour. Next, 55 mM IAM/50 mM ammonium bicarbonate was added to cover the gel slices and incubated for 1 hour at room temperature in the dark. After supernatant removal, 100 μL of 50 mM ammonium bicarbonate/acetonitrile (1:1, v/v) solution was added, followed by incubation in 1 mL of acetonitrile for 30 minutes. Enzyme digestion solution was then added to cover the gel slices, which were incubated on ice for 40 minutes. Subsequently, 200 μL of extraction solution (5% TFA-50% ACN-45% ddH_2_O) was added, followed by incubation in a 37°C water bath for 1 hour. The extract was centrifuged for 5 minutes, and the supernatant was transferred to a fresh tube. Five microliters of the sample were loaded for Nano LC-MS/MS Analysis.

The raw MS files were analyzed using Byonic, searching against a target protein database based on the species of the samples. Carbamidomethylation (C) was set as a fixed protein modification, while oxidation (M) and Acetyl(N-term) were set as variable modifications. N-glycan and O-glycan modifications were also considered. Chymotrypsin was specified as the enzyme, with a maximum of three missed cleavages allowed. The precursor ion mass tolerance was set to 20 ppm, and MS/MS tolerance was set to 0.02 Da. Only high-confidence identified peptides were selected for downstream protein identification analysis.

### Western blot and non-reducing page

For the SDS-PAGE, the purified proteins were resolved on 4–12% protein gel after being boiled in 5× SDS-PAGE loading buffer with Tris-Glycine-SDS Running buffer. The gel was stained with Coomassie Blue Staining Solution and imaged with Gel imager (Tanon).

For the Western blot, the purified proteins were transferred to a polyvinylidene fluoride (PVDF) membrane after SDS-PAGE, and blocked with 5% Non-fat Powdered Milk solutions for 2h at room temperature. After blocking, the membrane was incubated with anti-RBD rabbit serum diluted 1:1000 in PBST overnight at 4°C. After washing with PBST three times, the membrane was incubated for 1h with HRP-conjugated goat anti-rabbit secondary antibody. After another round of washing, the proteins were visualized using the ECL substrate.

For the non-reducing PAGE analysis [36], the purified proteins were mixed with 5×native sample loading buffer and resolved using precast 4–12% protein gel with Hepes-Tris Running buffer. And gel was stained with Coomassie Blue Staining Solution and imaged with Gel imager (Tanon).

### Mouse vaccination

mRNA vaccine immunization: A group of 6–8 weeks old female BALB/c mice were immunized i.m. with LNP/mRBD (10 μg, n = 6) or Placebo (PBS, n = 4). For boost immunization, each group of mice was boosted with an equal dose of LNP/mRBD on 14, 21, and 28 days after the primary injection. The serum was collected 14 days after booster immunization, And spleen tissues were collected at day 14 post-booster immunization to evaluate cellular immune responses by FCM as described below.

Recombinant RBD protein vaccine immunization: Female BALB/c mice (6–8 weeks old, n = 4 per group) were immunized by i.m. injection with two doses 28 days apart (study days 0 and 28) containing a dose range (2 or 20 μg/50 μL PBS) of recombinant protein with same volume Alum adjuvant. An adjuvant group served as non-immunized control. Serum was collected at 14, 28, and56 days post immunization to detect SARS-CoV-2 RBD specific IgG and neutralize antibody responses as described below.

### Deglycosylated RBD^WT^, RBD^M1^ and RBD^M2^ protein with PNGaseF

Combine 1–20 μg of glycoprotein, 1 μl of Glycoprotein Denaturing Buffer (10×) and H_2_O in a total reaction volume of 10 μl. Denature glycoprotein by heating reaction at 100°C for 10 minutes. Add 2 μl of GlycoBuffer 2 (10×), 2 μl of 10% NP-40, H_2_O, and 1 μl of PNGase F to make a total reaction volume of 20 μl, and incubate reaction at 37°C for 1 hour.

### SARS-CoV-2 RBD specific IgG by ELISA

Animal immune serum samples were heated at 56°C for 30 minutes before use. Briefly, 96-well microtiter plates were coated with 100ng/well of SARS-CoV-2 RBD protein (RBD^WT^, RBD^M1^, RBD^M2^, RBD^Deg-WT^, RBD^Deg-M1^, RBD^Deg-M2^). The plates were washed with PBST and blocked with 5% Non-fat Powdered Milk solutions. Serial 2-fold dilutions of mouse inactivated serum, starting at 1:100/1000, were added to the blocked plates and incubated at 37°C for 1 h. After washing with PBST, HRP-conjugated goat anti-mouse IgG (1:5000) was added to the plate and incubated for 1 h at 37°C. The plates were washed and developed using TMB (2-Component Microwell Peroxidase Substrate Kit), and the reaction was stopped using ELISA Stopping Solution. The plates were read at 450 nm wavelength using a plate reader. ELISA endpoint titers were defined as the highest reciprocal serum dilution that yielded an absorbance >2.1-fold over background values.

### Competitive ELISA

HRP-tagged S309 protein were labelled using Glue Activated Horseradish Peroxidase labeling kit (Solarbio). A 96-well microplate was coated with 100 ng of SARS-CoV-2 WT and XBB proteins per well separately. The plates were washed with PBST and blocked with a 5% non-fat powdered milk solution. A mixture of S309-HRP antibodies with diluted serum was prepared and added to the blocked wells. The plate was incubated at 37°C for 30 minutes. The plates were washed and developed using TMB. The reaction was stopped with ELISA Stopping Solution. The plates were measure at 450 nm wavelength using a microplate reader. Binding rate is the ratio of OD values of each serum group to the OD values of the PBS serum group.

### Pseudovirus-based neutralization assay

To determine the NT_50_ titer of immunized mouse serum, the 293T-hACE2 cells were seeded in 96-well plates (50,000 cells per well) and incubated for approximately 24 hours until reaching over 90% confluence in preparation for pseudovirus infection. The mouse serum was diluted 2-fold, starting at 1:10, and incubated with the SARS-CoV-2 pseudovirus (MOI~0.05) at 37°C for 1 hour. DMEM without serum was used as the negative control group. Then, the supernatant of 293T-hACE2 cells was removed, and a mixture of serum and pseudovirus was added to each well. After 48 hours, the luciferase activity, which reflects the degree of SARS-CoV-2 pseudovirus transfection, was measured. The 50% neutralization (NT_50_) titer was defined as the fold dilution that achieved more than 50% inhibition of pseudovirus infection compared with the control group.

### Authentic SARS-CoV-2 neutralization assay

Similarly, 10^4^ Vero cells/well were seeded 24 h before the infection in a 96-well plate (Costar). On the day of infection, the cells were washed twice with a cell culture medium. Sera from vaccinated mice were incubated at 56°C for 30 min and then two-fold (several times). Aliquots (40 μL) of diluted sera (from 20-fold to 5120-fold dilutions) were added to 50 μL of cell culture medium containing 100 tissue culture infective dose (TCID50) of Wild type, Delta, or Omicron virus strain (BA.5, XBB.1.16, JN.1) on a 96-well plate and incubated at 37°C for 2 h in 5% CO_2_. Virus serum mix was then added to cells in 96-well plates, and plates were incubated at 37°C with microscopic examination for cytopathic effect (CPE) after the 5-day incubation. The highest dilution of serum that showed inhibition activity of SARS-CoV-2 was estimated as the NT_50_ titer. NT assays were performed in triplicate with negative control sera. The threshold of neutralization activity is NT ≥ 4.

### Protection against SARS-CoV-2 XBB challenge in mice

Six- to eight-week-old specific pathogen-free female BALB/c mice (n = 5–6 per group) were respectively injected i.m. with two doses of mRNA vaccines and placebo at 21-day intervals. In BSL-3 facilities of Shenzhen Third People’s Hospital, all mice were intranasally challenged with 5×10^5^ TCID50 SARS-CoV-2 XBB.1.16 at 14 days post the second dose. Mice of each group were euthanized and lung were harvested at 3 d.p.i.

### Detection of SARS-CoV-2 RBD-specific IgG^+^ cell

Spleen were harvested on day 35 and SARS-CoV-2 WT-RBD-directed and XBB-RBD-directed IgG+ cell was screened via FCM. FITC-tagged WT-RBD protein were labelled using FITC conjugation kit (Sangon Biotech). And Biotin-tagged XBB-RBD protein were labelled using EZ-Link Sulfo-NHS-LC-Biotin (Thermo). Briefly, aqua Fixable Viability Stain 510 (BD) was first used to stain single cell suspensions for 30 min at 4°C. And, cells were co-incubated with 5ug RBD^WT^ and 5ug RBD^XBB^ for 30min at 4°C following washing. The following RBD^WT^-directed and RBD^XBB^-directed IgG^+^ cell staining panel of mouse-specific antibodies was then applied: RBD^WT^-FITC, RBD^XBB^-Biotin-Streptavidin (Streptavidin-PE, BD), IgG-PE/Cyanine7. the cells were acquired using LSR-Fortessa flow cytometer (BD), and the data were analyzed with FlowJo software. Sorted cells were IgG^+^ cells that were double-positive for SARS-CoV-2 RBD^WT^ and SARS-CoV-2 RBD^XBB^.

### Surface and intracellular cytokine staining

T cell assays were performed as previously described with minor modifications [37]. Functional responses of SARS-CoV-2 RBD specific CD4^+^ and CD8^+^ T cells in vaccinated animals were measured using peptide pools and intracellular cytokine staining assay. The SARS-CoV-2 RBD peptide pools consist mainly of 15-mer sequences with 11 amino acids overlap, which contains RBM and non-RBM two peptide pools. Splenocytes suspensions were prepared in PBS by mashing tissue against the surface of a 70-μm cell strainer. Two million cells were suspended in 100μL of RPMI 1640 medium with 10% FBS and stimulated with RBD peptide pools. The stimulated cells were incubated at 37°C in 5% CO_2_. After 1h of incubation, 2μg per well Protein Transport Inhibitor (contains Brefeldin A) was added and incubated for 8h at 37°C in 5% CO_2_. Cells were washed once with wash buffer and surface stained with Fixable Viability Dye, anti-CD3, anti-CD4, and anti-CD8, each conjugated to different fluorochrome for 45 min at 4°C. The stained cells were washed once with wash buffer and permeabilized with 250 μL Fixation/ Permeabilization solution for 20min at 4°C in dark. Permeabilized cells were washed once with 1× perm buffer and intracellular stained with anti-cytokine antibodies for 45 min at 4°C. Finally, the cells were washed with perm buffer twice and acquired using LSR-Fortessa flow cytometer (BD), and the data were analyzed with FlowJo software.

### Mouse immunization, cell fusion, and subclone amplifications

BALB/c mice (6–8 weeks, female) were immunized by subcutaneous injection of three recombinant proteins emulsified in an equal volume of complete Freund’s adjuvant. Incomplete Freund’s adjuvant was used for subsequent booster immunizations. Following five rounds of antigen immunization at 2-week intervals, spleens were collected from the mice. Splenocytes suspension were prepared and fused with SP2/0 myeloma cells and hybridoma cells were cultured in 96-well plates. After one week, the culture medium was replaced, and indirect ELISA was performed to assess the antibody titers in the cell supernatants, allowing for the selection of positive clones. Subsequently, limited dilution was employed to isolate single-cell subclones, which were then expanded and cultured.

BALB/c mice were intraperitoneally injected with 500 μL of incomplete Freund’s adjuvant. One week later, the expanded monoclonal cell was injected intraperitoneally into mice to induce antibody production. One week after the injection, ascitic fluid was collected from peritoneal cavity, the antibodies were precipitated by adding an equal volume of saturated ammonium sulfate solution. After centrifugation, the precipitate was resuspended in 1×PBS. The supernatant was subjected to affinity chromatography for purification. The purity of the antibodies was assessed by SDS-PAGE and were stored at -80°C.

### *In vivo* toxicity

BALB/c mice (n = 3) were immunized via the i.m. route with the mRNA vaccine candidate or PBS. After 24h post vaccination, all animals were sacrificed for tissue harvest and serum collection. IL-4 ELISA kits (Solarbio, SEKM-0005), IL-6 ELISA kits (Solarbio, SEKM-0007), IL-10 ELISA kits (Solarbio, SEKM-0010), IL-12 ELISA kits (Solarbio, SEKM-0012), IL-1β ELISA kits (Solarbio, SEKM-0002) IFN-γ ELISA kits (Solarbio, SEKM-0031) and TNF-α ELISA kits (Solarbio, SEKM-0034) were utilized for the analysis of immune-activated cytokines.

### Statistical analysis

All data were analyzed with GraphPad Prism. Data are presented as mean ± SEM in all experiments. Statistical significance was determined by unpaired, two-sided Student’s t-test for two-group comparisons, one-way analysis of variance (ANOVA) for comparisons of more than two groups, two-way ANOVA for comparisons of more than two groups with two or more items. (“*” represents p < 0.05, “**” represents p < 0.01, “***” represents p < 0.001, “****” represents p < 0.0001; “ns” represents not significant).

## Supporting information

S1 Fig Amino acid sequence of wide-type SARS-CoV-2 RBD and glycosylated RBDs. RBD sequences are shown in black, signal peptide sequences are shown in pink, T4f sequences are shown in green, Glycan-masking mutations are marked in light red in RBD-M1 and M2, respectively. “*” represents termination codon.
(TIF)

S2 Fig Total ion chromatogram for RBD^M1^ protein glycosylation site (type) detection and glycans NHFAGNa analysis. (TIF)

S3 Fig Total ion chromatogram for RBD^M2^ protein glycosylation site (type) detection and glycans NHFAGNa analysis. (TIF)

S4 Fig Surface representation of wild-type SARS-CoV-2 and glycosylated RBD^M1^ and RBD^M2^ shown as green and other coloured spheres using the FoldX algorithm. (TIF)

S5 Fig Transcytosis capacity of LNP. (A) Diagram of transcytosis. (B-C) Transcytosis efficiency of LNP/Cy5-nucleic acid in HEK-293T cells measured by FCM and quantitative analysis. (D-E) Transcytosis capacity of LNP/mRNA complexes in HEK-293T cells was imaged by CLSM and quantification of Cy5 signaling. The Cy5 signals were observed in (i), (ii), and (iii). The Cy5 signal in (ii) emanated from cells in (i) and subsequently underwent endocytosis by (ii). The Cy5 signal in (iii) originated from cells in (ii) and was absorbed by (iii), scale bar: 10 μm.
(TIF)

S6 Fig The inflammatory reactions observed 24 hours post-vaccination. (TIF)

S7 Fig Immunohistochemistry (IHC) analysis of lung after XBB.1.16 challenge. (TIF)

S8 Fig Expression, purification and binding activities characterization of SARS-CoV-2 monoclonal antibodies. (A) SDS-PAGE of purified antibodies. (B) EC50 binding reaction value of each antibody to the antigen.
(TIF)

S1 Table Analysis of Glycosylation site in RBD^M1^ protein. (DOCX)

S2 Table Analysis of Glycosylation site in RBD^M2^ protein. (DOCX)

S1 Data Raw data of Figures. (XLSX)

S2 Data Raw data of Western Blot. (PPTX)
