# Supplementary figures and images for: Development of Glycan-masked SARS-CoV-2 RBD vaccines against SARS-related coronaviruses
Source: PLoS Pathog. 2024 Sep 26;20(9):e1012599. doi: 10.1371/journal.ppat.1012599 (PMC11460674; doi:10.1371/journal.ppat.1012599)

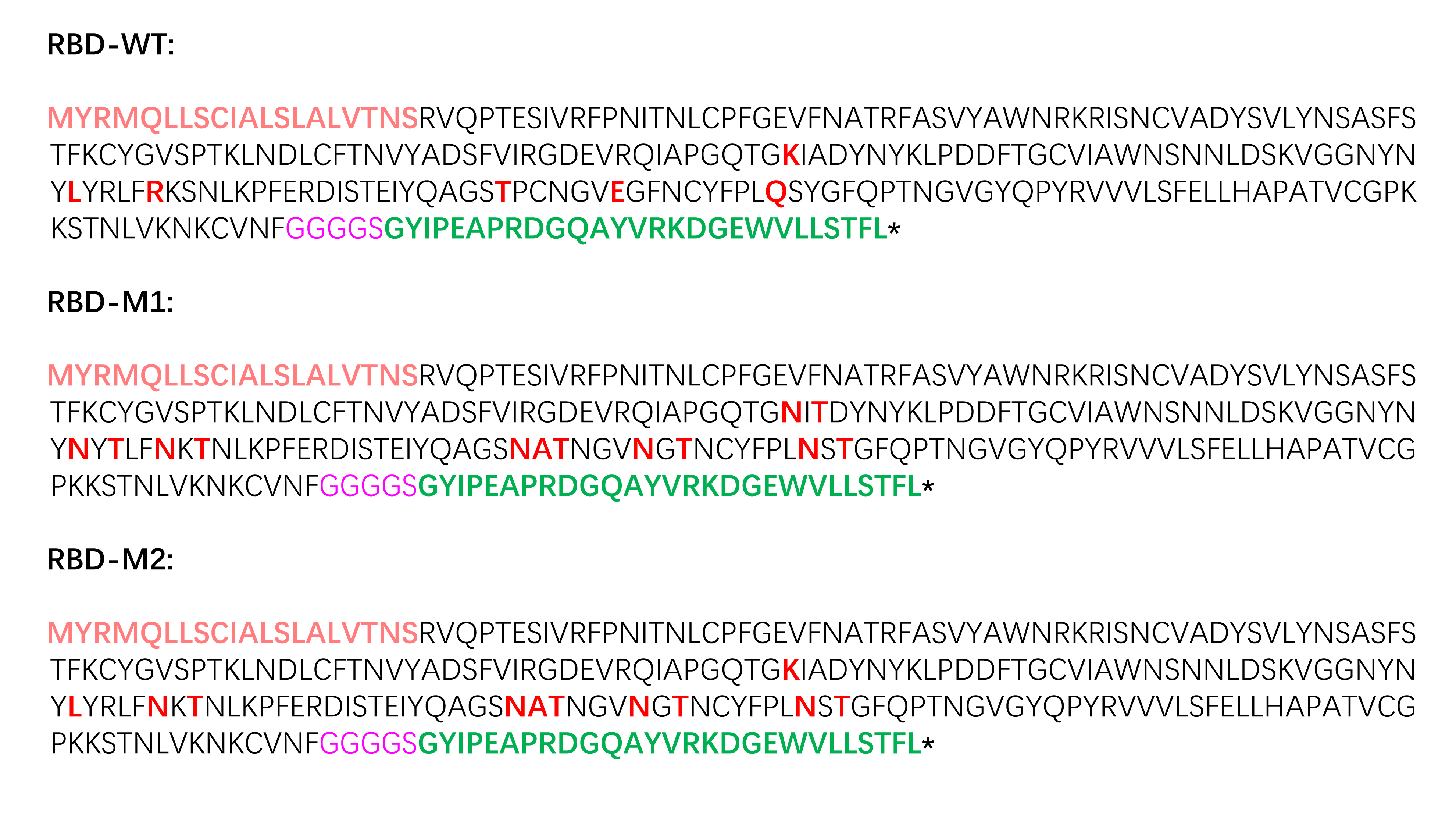

Supplement: S1 Fig — RBD sequences are shown in black, signal peptide sequences are shown in pink, T4f sequences are shown in green, Glycan-masking mutations are marked in light red in RBD-M1 and M2, respectively. “*” represents termination codon. (TIF) [file ppat.1012599.s001.tif]

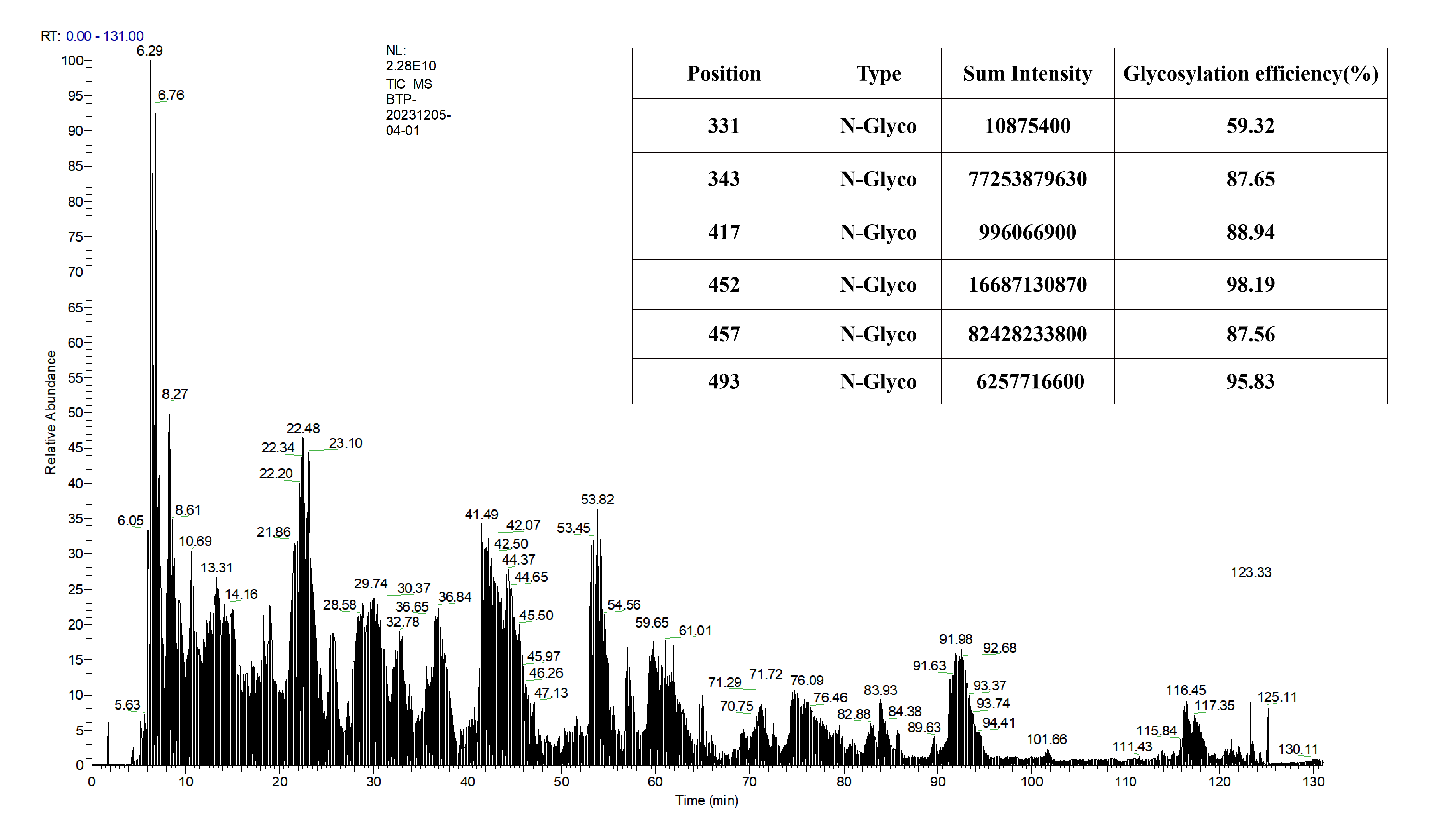

Supplement: S2 Fig — (TIF) [file ppat.1012599.s002.tif]

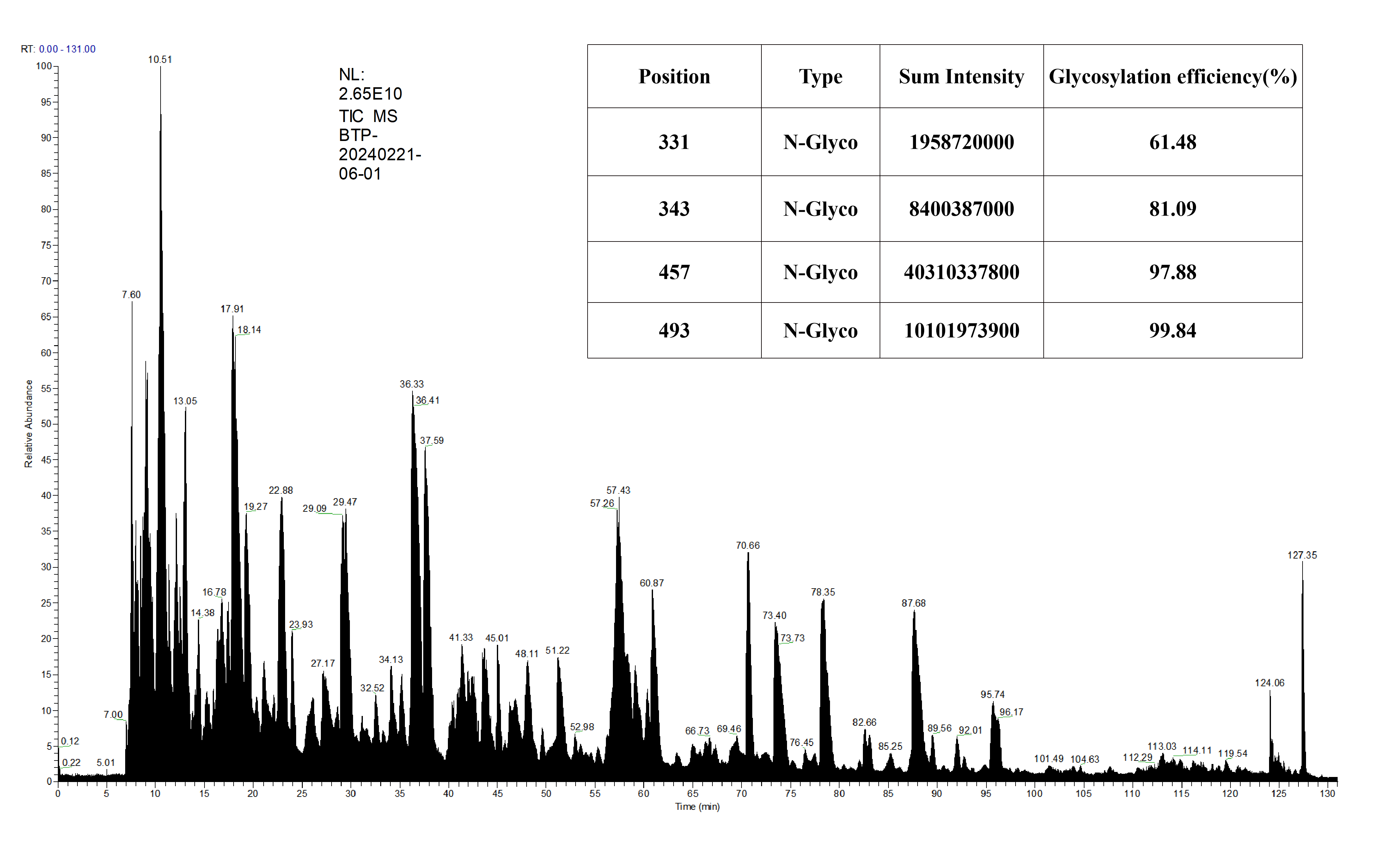

Supplement: S3 Fig — (TIF) [file ppat.1012599.s003.tif]

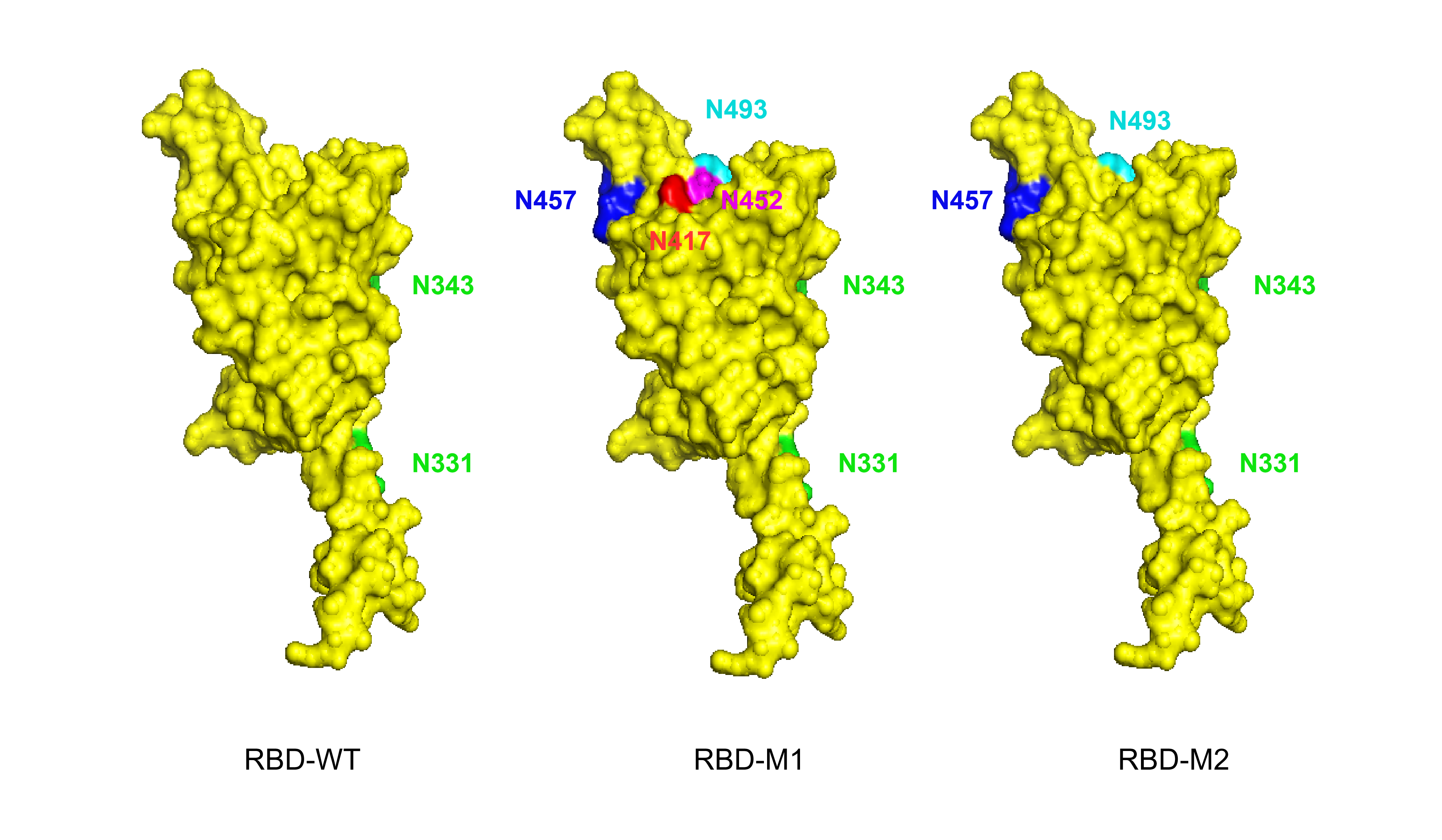

Supplement: S4 Fig — (TIF) [file ppat.1012599.s004.tif]

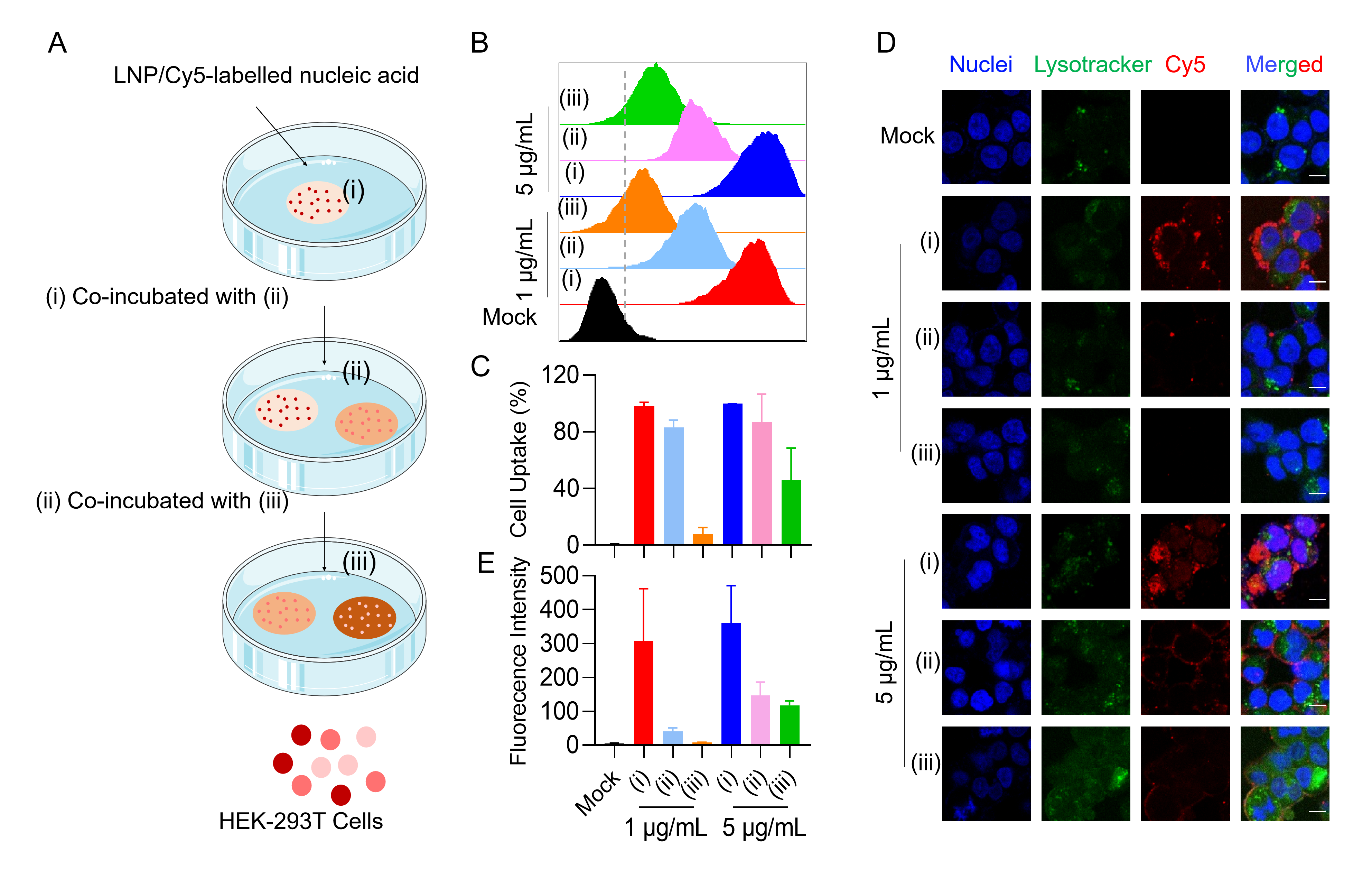

Supplement: S5 Fig — (A) Diagram of transcytosis. (B-C) Transcytosis efficiency of LNP/Cy5-nucleic acid in HEK-293T cells measured by FCM and quantitative analysis. (D-E) Transcytosis capacity of LNP/mRNA complexes in HEK-293T cells was imaged by CLSM and quantification of Cy5 signaling. The Cy5 signals were observed in (i), (ii), and (iii). The Cy5 signal in (ii) emanated from cells in (i) and subsequently underwent endocytosis by (ii). The Cy5 signal in (iii) originated from cells in (ii) and was absorbed by (iii), scale bar: 10 μm. (TIF) [file ppat.1012599.s005.tif]

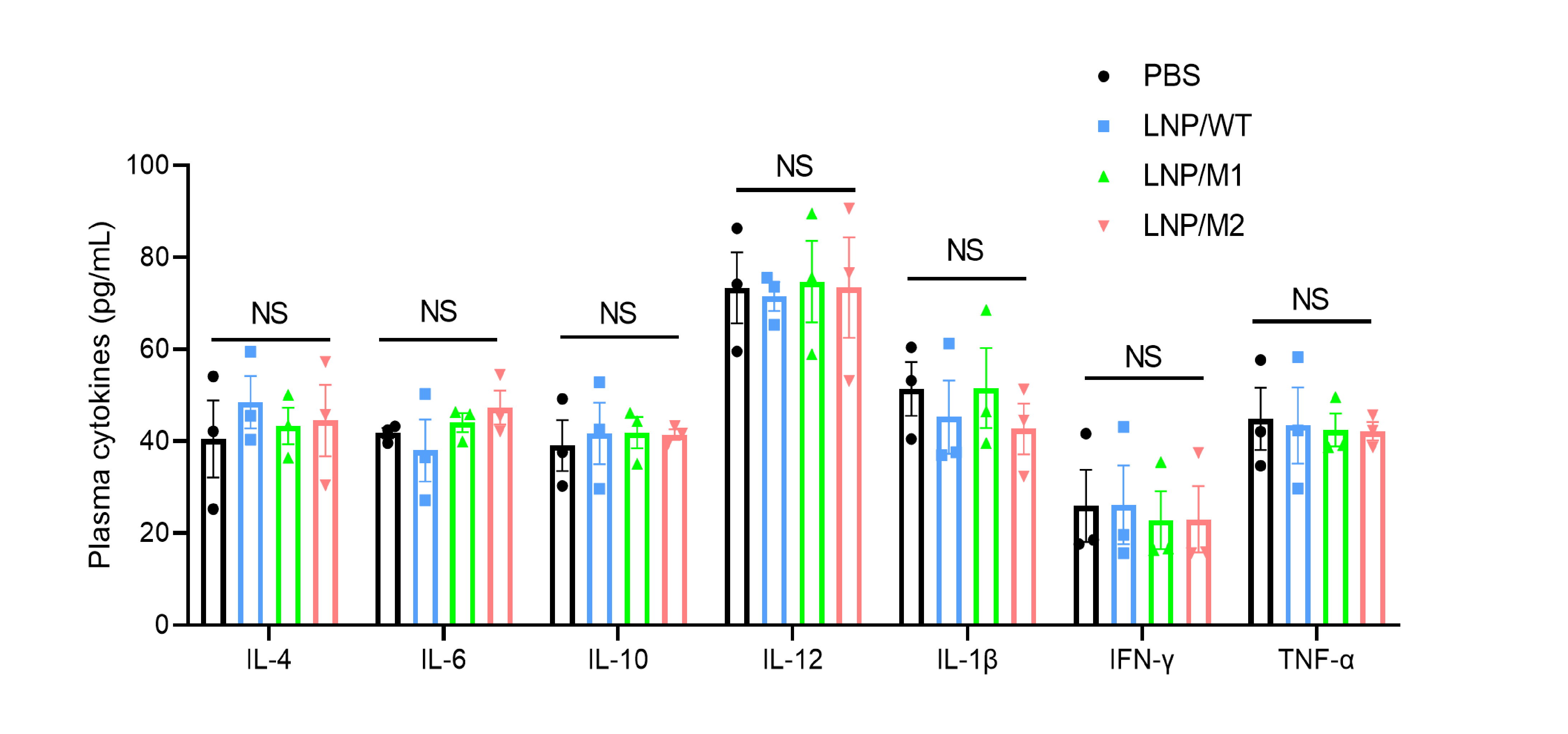

Supplement: S6 Fig — (TIF) [file ppat.1012599.s006.tif]

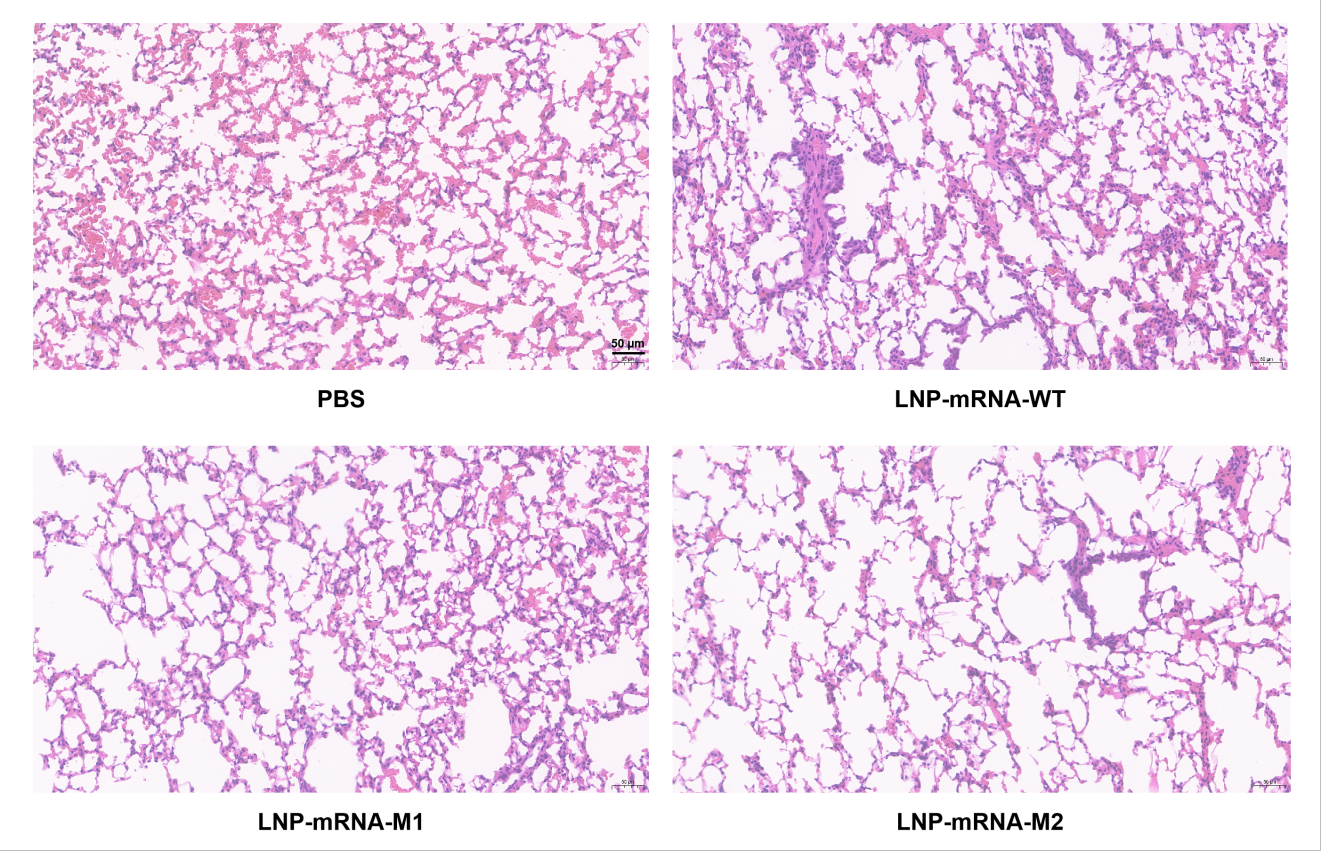

Supplement: S7 Fig — (TIF) [file ppat.1012599.s007.tif]

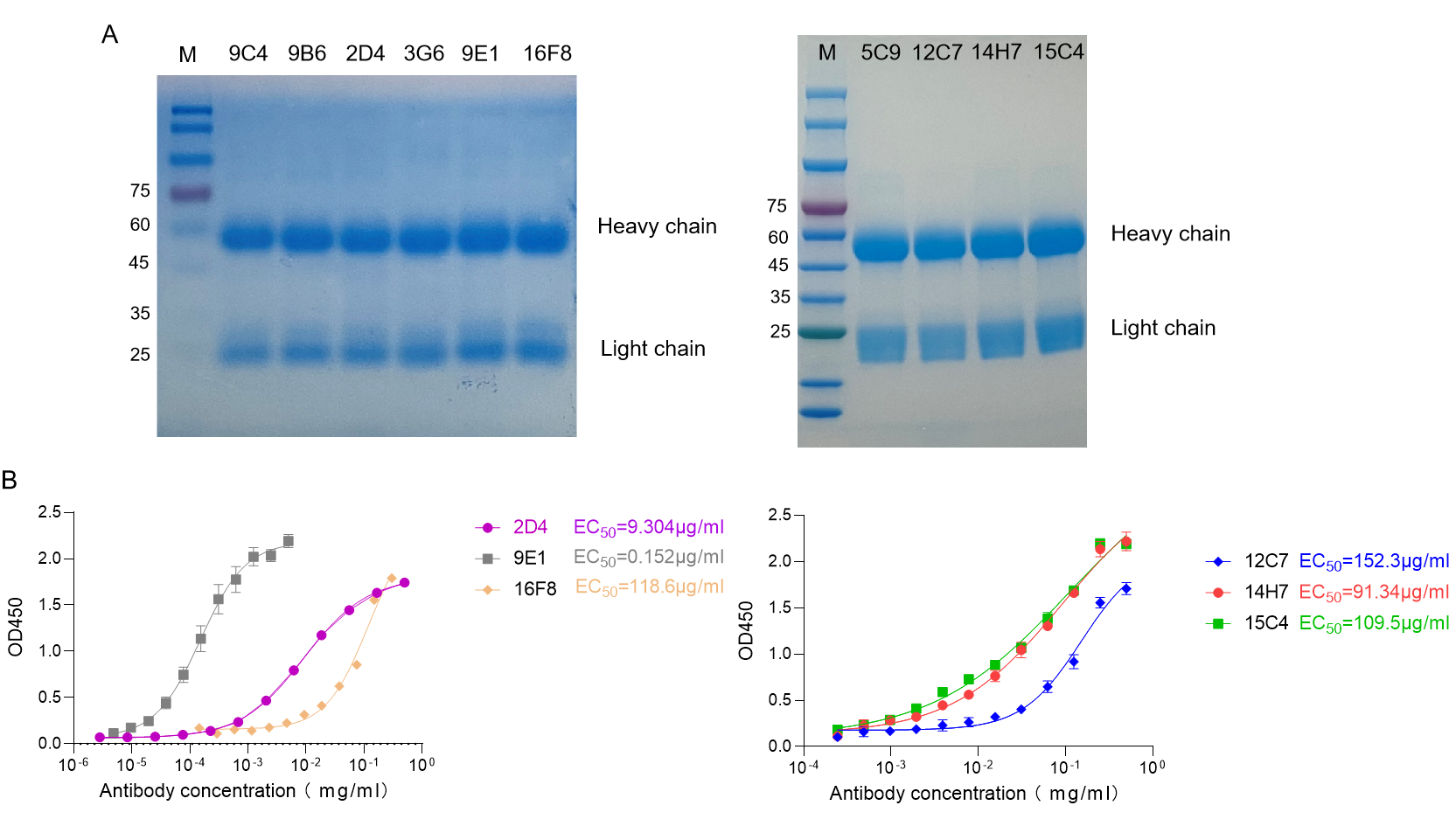

Supplement: S8 Fig — (A) SDS-PAGE of purified antibodies. (B) EC50 binding reaction value of each antibody to the antigen. (TIF) [file ppat.1012599.s008.tif]

## Slide 1
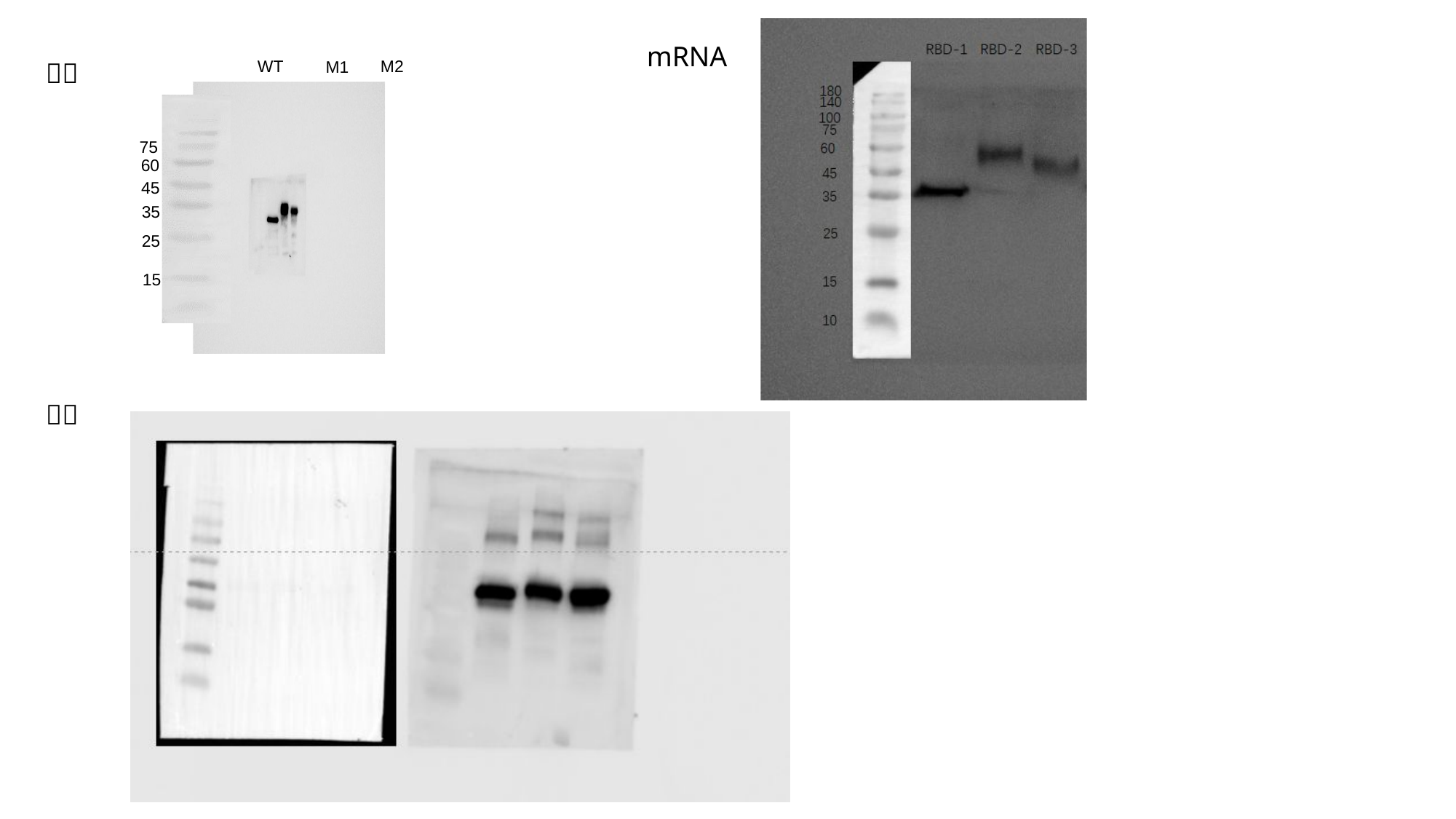

mRNA
蛋白
WT
M2
M1
45
75
60
35
25
15
脱糖

Supplement: S2 Data — (PPTX) [file ppat.1012599.s012.pptx]
