# Supplementary material for: Development of Glycan-masked SARS-CoV-2 RBD vaccines against SARS-related coronaviruses
Source: PLoS Pathog. 2024 Sep 26;20(9):e1012599. doi: 10.1371/journal.ppat.1012599 (PMC11460674; doi:10.1371/journal.ppat.1012599)
Supplement: S1 Table — (DOCX) [file ppat.1012599.s009.docx]

**S1_Table. Analysis of Glycosylation site in RBD^M1^** **protein.**

| Type | Position | Peptide | Glycans  NHFAGNa | Modification Type | Observed (M+H) | Calc.  mass (M+H) | Mass error  (ppm) | Starting  position | Score | Intensity |
| --- | --- | --- | --- | --- | --- | --- | --- | --- | --- | --- |
| N-Glyco | 331 | F.PN[+2272.846]ITNL.C | HexNAc(8)Hex(4) | N[+2273] | 2944.212 | 2944.2185 | -2.2 | 32 | 19.19 | 5437700 |
|  | 343 | F.N[+1054.370]ATRF.A | HexNAc(2)Hex(4) | N[+1054] | 1662.6819 | 1662.6851 | -1.9 | 45 | 389.23 | 231770000 |
|  | 343 | F.GEVFN[+1216.423]ATRF.A | HexNAc(2)Hex(5) | N[+1216] | 2256.9335 | 2256.9388 | -2.4 | 41 | 89.16 | 4363300 |
|  | 417 | F.VIRGDEVRQIAPGQTGN[+349.137]ITDY.N | HexNAc(1)Fuc(1) | N[+349] | 2651.3119 | 2651.3159 | -1.5 | 103 | 351.25 | 182100000 |
|  | 452 | Y.NYN[+203.079]YTLF.N | HexNAc(1) | N[+203] | 1137.5076 | 1137.5099 | -2 | 152 | 522.04 | 12631000 |
|  | 452 | Y.NYN[+1727.613]Y.T | HexNAc(3)Hex(6)Fuc(1) | N[+1728] | 2300.8395 | 2300.8433 | -1.7 | 152 | 350.71 | 33467000 |
|  | 452 | Y.N[+1606.587]YTLF.N | HexNAc(4)Hex(4)Fuc(1) | N[+1607] | 2263.9135 | 2263.9109 | 1.1 | 154 | 283.46 | 15307000 |
|  | 452 | Y.NYN[+1565.560]YTL.F | HexNAc(3)Hex(5)Fuc(1) | N[+1566] | 2352.9352 | 2352.9222 | 5.5 | 152 | 272.52 | 68817000 |
|  | 452 | Y.N[+1768.640]Y.T | HexNAc(4)Hex(5)Fuc(1) | N[+1769] | 2064.7609 | 2064.7636 | -1.3 | 154 | 234.63 | 338740000 |
|  | 452 | Y.N[+2110.793]YTL.F | HexNAc(8)Hex(3) | N[+2111] | 2621.0494 | 2621.0493 | 0 | 154 | 36.77 | 13050000 |
|  | 457 | F.N[+203.079]KTNLKPF.E | HexNAc(1) | N[+203] | 1164.6248 | 1164.6259 | -1 | 159 | 675.82 | 802960000 |
|  | 457 | F.N[+1768.640]KTNL.K | HexNAc(4)Hex(5)Fuc(1) | N[+1769] | 2357.9699 | 2357.9699 | 0 | 159 | 344.12 | 318020000 |
|  | 457 | L.FN[+1606.587]KTNLKPF.E | HexNAc(4)Hex(4)Fuc(1) | N[+1607] | 2715.1987 | 2715.2016 | -1.1 | 158 | 148.51 | 9686400 |
|  | 457 | L.FN[+1971.719]KTNL.K | HexNAc(5)Hex(5)Fuc(1) | N[+1972] | 2708.1076 | 2708.1177 | -3.7 | 158 | 67.67 | 15799000 |
|  | 493 | Y.FPLN[+1768.640]STGF.Q | HexNAc(4)Hex(5)Fuc(1) | N[+1769] | 2651.0707 | 2651.0751 | -1.7 | 192 | 490.82 | 209640000 |
|  | 493 | L.N[+349.137]STGF.Q | HexNAc(1)Fuc(1) | N[+349] | 874.3664 | 874.3676 | -1.4 | 195 | 456.21 | 6692900 |
|  | 493 | Y.FPLN[+1768.640]STGFQPTNGVGY.Q | HexNAc(4)Hex(5)Fuc(1) | N[+1769] | 3467.4406 | 3467.4517 | -3.2 | 192 | 129.71 | 24940000 |
|  | 493 | L.N[+1216.423]STGFQPTNGVGY.Q | HexNAc(2)Hex(5) | N[+1216] | 2558.0197 | 2558.0298 | -4 | 195 | 95.54 | 8034800 |
|  | 493 | F.PLN[+1403.507]STGF.Q | HexNAc(3)Hex(4)Fuc(1) | N[+1404] | 2138.8769 | 2138.8745 | 1.1 | 193 | 11.7 | 2965600 |
