# Supplementary material for: Development of Glycan-masked SARS-CoV-2 RBD vaccines against SARS-related coronaviruses
Source: PLoS Pathog. 2024 Sep 26;20(9):e1012599. doi: 10.1371/journal.ppat.1012599 (PMC11460674; doi:10.1371/journal.ppat.1012599)
Supplement: S2 Table — (DOCX) [file ppat.1012599.s010.docx]

| Type | Position | Peptide | Glycans  NHFAGNa | Modification Type | Observed  (M+H) | Calc.  mass (M+H) | Mass error  (ppm) | Starting position | Score | Intensity |
| --- | --- | --- | --- | --- | --- | --- | --- | --- | --- | --- |
| N-Glyco | 331 | F.PN[+2272.846]ITNL.C | HexNAc(8)Hex(4) | N[+2273] | 1876.6541 | 1876.4263 | 0.1 | 36 | 91.37 | 25471862 |
|  | 343 | F.N[+1216.423]ATRF.A | HexNAc(2)Hex(5) | N[+1216] | 1824.7383 | 1824.7379 | 0.2 | 45 | 296.41 | 165660000 |
|  | 343 | F.N[+203.079]ATRFASVY.A | HexNAc(1) | N[+203] | 1231.5889 | 1231.5953 | -5.2 | 45 | 160.31 | 17280000 |
|  | 457 | F.N[+203.079]KTNLKPF.E | HexNAc(1) | N[+203] | 1164.6266 | 1164.6259 | 0.6 | 159 | 581.27 | 455360000 |
|  | 457 | L.FN[+730.264]KTNLKPF.E | HexNAc(2)Hex(2) | N[+730] | 1838.8752 | 1838.8793 | -2.3 | 158 | 206.04 | 16771000 |
|  | 457 | F.N[+2133.772]KTNL.K | HexNAc(5)Hex(6)Fuc(1) | N[+2134] | 2723.1321 | 2723.1021 | 11 | 159 | 146.05 | 16407000 |
|  | 457 | F.N[+2701.983]KTNLKPFERDISTEIYQAGSNATNGVNGTNC[+57.021]Y.F | HexNAc(7)Hex(7)Fuc(1) | N[+2702] | 6378.7642 | 6378.7129 | 8 | 159 | 76.65 | 105810000 |
|  | 493 | Y.FPLN[+1768.640]STGF.Q | HexNAc(4)Hex(5)Fuc(1) | N[+1769] | 2651.0768 | 2651.0751 | 0.6 | 192 | 444.5 | 141680000 |
|  | 493 | L.N[+1768.640]STGF.Q | HexNAc(4)Hex(5)Fuc(1) | N[+1769] | 2293.8724 | 2293.8699 | 1.1 | 195 | 391.8 | 69181000 |
|  | 493 | F.ERDISTEIYQAGSNATNGVNGTNC[+57.021]YFPLN[+1702.581]STGF.Q | HexNAc(2)Hex(8) | N[+1703] | 5299.2926 | 5300.2 | 18.1 | 167 | 30 | 154660000 |

**S2_Table. Analysis of Glycosylation site in** **RBD^M2^ protein.**
